# Searching for Drug Synergy Against Cancer Through Polyamine Metabolism Impairment: Insight Into the Metabolic Effect of Indomethacin on Lung Cancer Cells

**DOI:** 10.3389/fphar.2019.01670

**Published:** 2020-02-28

**Authors:** Freddy López-Contreras, Matías Muñoz-Uribe, Jorge Pérez-Laines, Laura Ascencio-Leal, Andrés Rivera-Dictter, Antonia Martin-Martin, Rafael A. Burgos, Pablo Alarcon, Rodrigo López-Muñoz

**Affiliations:** ^1^ Facultad de Ciencias Veterinarias, Instituto de Farmacología y Morfofisiología, Universidad Austral de Chile, Valdivia, Chile; ^2^ Facultad de Ciencias Veterinarias, Escuela de Graduados, Universidad Austral de Chile, Valdivia, Chile

**Keywords:** indomethacin, spermidine/spermine-N1-acetyltransferase, polyamine, non-small cell lung cancer, metabolism

## Abstract

Non-small cell lung cancer (NSCLC) is the most lethal and prevalent type of lung cancer. In almost all types of cancer, the levels of polyamines (putrescine, spermidine, and spermine) are increased, playing a pivotal role in tumor proliferation. Indomethacin, a non-steroidal anti-inflammatory drug, increases the abundance of an enzyme termed spermidine/spermine-N^1^-acetyltransferase (SSAT) encoded by the *SAT1* gene. This enzyme is a key player in the export of polyamines from the cell. The aim of this study was to compare the effect of indomethacin on two NSCLC cell lines, and their combinatory potential with polyamine-inhibitor drugs in NSCLC cell lines. A549 and H1299 NSCLC cells were exposed to indomethacin and evaluations included *SAT1* expression, SSAT levels, and the metabolic status of cells. Moreover, the difference in polyamine synthesis enzymes among these cell lines as well as the synergistic effect of indomethacin and chemical inhibitors of the polyamine pathway enzymes on cell viability were investigated. Indomethacin increased the expression of *SAT1* and levels of SSAT in both cell lines. In A549 cells, it significantly reduced the levels of putrescine and spermidine. However, in H1299 cells, the impact of treatment on the polyamine pathway was insignificant. Also, the metabolic features upstream of the polyamine pathway (i.e., ornithine and methionine) were increased. In A549 cells, the increase of ornithine correlated with the increase of several metabolites involved in the urea cycle. Evaluation of the levels of the polyamine synthesis enzymes showed that ornithine decarboxylase is increased in A549 cells, whereas S-adenosylmethionine-decarboxylase and polyamine oxidase are increased in H1299 cells. This observation correlated with relative resistance to polyamine synthesis inhibitors eflornithine and SAM486 (inhibitors of ornithine decarboxylase and S-adenosyl-L-methionine decarboxylase, respectively), and MDL72527 (inhibitor of polyamine oxidase and spermine oxidase). Finally, indomethacin demonstrated a synergistic effect with MDL72527 in A549 cells and SAM486 in H1299 cells. Collectively, these results indicate that indomethacin alters polyamine metabolism in NSCLC cells and enhances the effect of polyamine synthesis inhibitors, such as MDL72527 or SAM486. However, this effect varies depending on the basal metabolic fingerprint of each type of cancer cell.

## Introduction

According to the World Health Organization, lung cancer was the leading cause of cancer-related death worldwide in 2018 (Bray et al., 2018). Lung cancer is sub-classified into non-small cell lung cancer (NSCLC) and small cell lung cancer, which account for 85 and 15% of cases, respectively (Devita et al., 2015). NSCLC is associated with the poorest prognosis and generally diagnosed at the advanced stages of disease (Goldstraw et al., 2016). Moreover, the most effective treatment currently available increases the 5-year survival in merely 15% of patients. Notably, all other available regimens increase survival in 1–5% of patients (Artal Cortes et al., 2015).

Natural polyamines (putrescine, spermidine, and spermine) are essential factors for the proliferation and survival of eukaryotic cells. Polyamines are related to multiple cell functions, including the regulation of gene expression, cell cycle modulation, and maintenance of nucleic acid and membrane stability (Casero and Marton, 2007; Pegg and Casero, 2011). It has been shown that tumor cells possess higher levels of polyamines, compared with their surrounding tissues (Agostinelli et al., 2010). Indeed, increased levels of polyamines in the plasma and urine are indicative of poor prognosis in cancer patients, including those with NSCLC (Kato et al., 2014).

This increase in the concentration of polyamines in cancer cells is related to the upregulation of polyamine synthesis and uptake from the external milieu, and downregulation of polyamine catabolism and acetylation for exportation (Gerner and Meyskens, 2004). The following two enzymatic processes are recognized as rate-limiting steps in polyamine synthesis: the conversion of ornithine to putrescine [mediated by the ornithine-decarboxylase (ODC) enzyme] and the decarboxylation of S-adenosyl-L-methionine [mediated by the S-adenosyl-L-methionine decarboxylase (AMD1)], which is essential for spermidine and spermine synthesis (Casero and Marton, 2007). Although the synthesis of polyamines is well documented, the polyamine uptake (mediated by the polyamine transport system) remains poorly understood (Poulin et al., 2012).

On the other hand, depletion of polyamines can be achieved by catabolism or exportation in its acetylated form. In this setting, spermine is back-converted to spermidine by the action of the spermine oxidase (SMOX), whereas spermine and spermidine are acetylated by the action of the spermidine/spermine-N1-acetyltransferase (SSAT) enzyme (encoded by the *SAT1* gene), for further extrusion from the cell *via* the SCL32 transporter (Casero and Marton, 2007). SSAT activity has been highlighted as a potential target of chemotherapy against tumor cells, and this enzyme can be induced by several stimuli, such as steroidal hormones, calcitriol, catecholamines, and insulin-like growth factor 1 (Pegg, 2008). Consequently, the depletion of spermidine and spermine through the overexpression of SSAT induces cell cycle arrest in HeLa cells (Mandal et al., 2013). In addition, certain non-steroidal anti-inflammatory drugs (NSAIDs) have been described as inducers of *SAT1* expression. For instance, heteroaryl-acetic acid NSAIDs (e.g., indomethacin and sulindac) have been linked to an increase in the levels of SSAT, promoting the extrusion of acetylated polyamines from the cell, and exerting an antiproliferative effect on colon cancer cells (Turchanowa et al., 2001; Babbar et al., 2003).

Despite the detrimental effect of SSAT increase on tumor cells, the increase in polyamine acetylation can be reverted by the polyamine oxidase (PAOX) enzyme. Thus, PAOX is considered an alternative or “rescue” pathway of putrescine and spermidine synthesis, using acetylated spermidine and spermine as substrates, respectively (Casero et al., 2018).

A large body of evidence supports the use of NSAIDs as a chemopreventive tool in patients at high risk of colon cancer (Mohammed et al., 2018; Umezawa et al., 2019). One of the modes of action proposed for NSAIDs in this setting is overexpression of the SSAT enzyme (Turchanowa et al., 2001; Babbar et al., 2003; Babbar et al., 2006a). This supports the clinical use of NSAIDs in combination with difluoromethylornithine (DFMO), an ODC inhibitor (Meyskens et al., 2008; Thompson et al., 2010; Lynch et al., 2016).

Indomethacin, one of the first NSAIDs approved for the treatment of pain (Lucas, 2016) has also shown an *in vivo* effect on the growth of breast cancer in rats (Fulton, 1984). More interestingly, it increases survival in patients with several types of cancer at advanced stages (Lundholm et al., 1994). However, there is limited information regarding the potential role of indomethacin in the impairment of polyamine metabolism in NSCLC.

The aim of this study was to evaluate the effect of indomethacin on the expression of *SAT1*, SSAT levels, and polyamine metabolism in two NSCLC cells lines with different genetic and metabolic background. Moreover, the synergistic effect of indomethacin with different inhibitors of the polyamine synthesis enzymes (i.e., ODC, AMD1, PAOX, and SMOX) on the viability of these NSCLC cells was investigated.

## Materials and Methods

### Cell Culture

The human NSCLC cell lines A549 (CCL-185™) and H1299 (CRL-5803™) were purchased from the American Type Culture Collection (ATCC, Manassas, VA, USA). The cells were cultured in humidified air and 5% CO_2_ using Roswell Park Memorial Institute (RPMI) 1640 (for H1299 cells) or Dulbecco’s modified Eagle’s medium F-12K (for A549 cells) culture media, supplemented with 10% fetal bovine serum (Cat #04-001-1A, US Origin; Biological Industries, Cromwell, CT, USA) and antibiotics (penicillin 100 U/ml and streptomycin 100 μg/ml). We also verified that fetal bovine serum does not contain polyamines, as determined through high-performance liquid chromatography (**Supplementary Methods**, **Supplementary Results** and **Supplementary Figure S1**). The normal epithelial lung cell line BEAS-2B (Cat #95102433) was purchased from the European Collection of Authenticated Cell Cultures (Salisbury, UK) and grown in LHC-9 medium (Thermo Fisher Scientific, Rockford, IL, USA). All cells were used for ≤20 passages.

### Drugs and Experimental Design

Indomethacin (purity 100%), MDL72725 (purity 100%), and eflornithine (DFMO, purity 100%) were purchased from Cayman Chemical (Ann Arbor, MI, USA), while SAM486 (purity >98%) was purchased from MedChemExpress (Monmouth Junction, NJ, USA). All drugs were dissolved in dimethyl sulfoxide. For all experiments, the maximal concentration of dimethyl sulfoxide in the culture medium was 0.5%, which did not influence cell viability. For the western blotting, quantitative reverse transcription-polymerase chain reaction (qRT-PCR), and metabolomic studies, the cells were exposed to 0.5 and 1 mM of indomethacin for 24 h, as previously described (Sanchez-Alcazar et al., 2003; Zhang et al., 2011). For the cell viability and combination studies, the cells were exposed to six decreasing concentrations of each drug for 96 h.

### Ribonucleic Acid Extraction and Quantitative Reverse Transcription-Polymerase Chain Reaction

H1299 and A549 cells were seeded in six-well plates (5 × 10^5^ cells per well) and allowed to attach overnight. The cells were subsequently treated with indomethacin for 24 h. After the treatment, RNA extraction was performed using the E.Z.N.A.^®^ Total RNA kit I (Omega Bio-Tek, Norcross, GA, USA). The RNA obtained was treated with the Turbo DNA-free^®^ deoxyribonuclease (DNAse) kit (Thermo Fisher Scientific, Vilnius, Lithuania), followed by complementary DNA (cDNA) synthesis using the AffinityScript™ Quantitative PCR (qPCR) cDNA synthesis kit (Agilent Technologies, Cedar Creek, TX, USA). Subsequently, the cDNA was used as a template for the qRT-PCR reaction. For this purpose, the Brilliant II SYBR^®^ Green qPCR Master Mix kit (Agilent Technologies Cedar Creek, TX, USA) was used. The qRT-PCR reaction was performed in a qPCR StepOne™ (Applied Biosystems, Waltham, MA USA) equipment using specifically designed primers for *sat1* messenger RNA (mRNA) (forward 5’-CAGTGACATACTGCGGCTGAT-3’ and reverse 3’-TTTCGGCACTTCTGCAACCA-5’), and the *glyceraldehyde 3-phosphate dehydrogenase* mRNA (*gapdh*, forward 5’-GGAGCGAGATCCCTCCAAAAT-3’ and reverse 3’-R GGCTGTTGTCATACTTCTCATGG-5’) housekeeping gene. The configuration of the PCR amplification involved an initial denaturation step of 10 min at 95°C, followed by 40 cycles: 1 min of denaturation at 95°C, 30 s of annealing at 55°C, and extension for 30 s at 72°C.

### Western Blotting

H1299, A549, and BEAS-2B cells were seeded in six-well plates (1 × 10^6^ cells per well) and allowed to attach for 24 h. For the measurement of its effect, H1299 and A249 cells were exposed to indomethacin for 24 h. Cell lysates were prepared using Pierce™ radioimmunoprecipitation assay (RIPA) buffer (Thermo Fisher Scientific, Rockford, IL, USA) containing 25 mM Tris–hydrochloride (pH 7.6), 150 mM sodium chloride, 1% NP-40, 1% sodium deoxycholate, and 0.1% sodium dodecyl sulfate (SDS), supplemented with Halt™ protease inhibitor cocktail (Thermo Fisher Scientific, Rockford, IL, USA). Samples were placed on ice for 10 min and centrifuged for 15 min at 15,000 *g*. Total protein concentration was determined using the protein assay dye reagent concentrate (Bio-Rad Laboratories, Hercules, CA, USA). Subsequently, 80 μg of total protein were denatured at 95°C for 5 min and resolved using 15% SDS-polyacrylamide gel electrophoresis at 25 mA. Proteins were electrotransferred to a Protran™ pure nitrocellulose membrane 0.45 μm (PerkinElmer, Waltham, MA, USA) at 450 mA for 1 h. The membrane was blocked using 5% non-fat milk in Tris-buffered saline containing 0.1% Tween 20 (TBST, pH 7.4) for 1 h at room temperature and washed with TBST. The membranes were incubated with primary antibodies against SSAT (Cat #61586, 1:2,000), ODC (Cat# sc-398116, 1:2,000), AMD1 (Cat# sc-166970, 1:2,000), PAOX (Cat# ab84951, 1:2,000), SMOX (Cat #SAB1101510), or vinculin (Cat # sc-73614, 1:10,000), as loading control, in 5% non-fat milk in 0.1% TBST at 4°C overnight. The membranes were washed with 0.1% TBST and incubated with a secondary anti-rabbit immunoglobulin G conjugated to horseradish peroxidase (IgG-HRP) (Cat# sc-2004 1: 5,000) or anti-mouse IgG-HRP (Cat# 115-035-003, 1: 5,000) antibody in 5% milk in 0.1% TBST. The SSAT antibody was purchased from Cell Signaling Technologies (Danvers, MA, USA), the ODC, AMD1, vinculin, and anti-rabbit IgG-HRP antibodies were purchased from Santa Cruz Biotechnology (Dallas, TX, USA), the SMOX antibody was purchased from Sigma-Aldrich (St. Louis, MO, USA), and the anti-mouse IgG-HRP was purchased from Jackson ImmunoResearch Laboratories (West Grove, PA, USA). Following incubation, the membranes were washed with 0.1% TBST. Blots were imaged using an Odyssey^®^ Fc Western blot scanner (LI-COR Biosciences, Lincoln, NE, USA). Western blotting quantitation was performed using the Image Studio Lite version 5.0 (LI-COR Biosciences).

### Sample Preparation for Gas Chromatography-Mass Spectrometry Metabolomics

H1299 and A549 cells were seeded in Petri dishes at a density of 6 × 10^6^ cells and allowed to attach for 24 h. Subsequently, they were exposed to indomethacin 1 mM for 24 h. Metabolites were extracted using 1 ml of cold acetonitrile:isopropanol:water (3:3:2) mixture, containing 10 μl deuterated myristic acid d-27 (Sigma-Aldrich) in hexane (0.75 mg/ml) as an internal standard. Samples were vortexed for 10 s, maintained at 4°C for 5 min on a shaker, and centrifuged at 14,000 *g* for 2 min at room temperature. After centrifugation, 450 μl of the supernatant were evaporated to dryness using a SpeedVac™ concentrator (Savant^®^ SPD131DDA; Thermo Fisher Scientific, Waltham, MA, USA). Subsequently, 450 μl of cold acetonitrile:water (50:50) were added and vortexed for 10 s, centrifuged at 14,000 *g* for 2 min at room temperature, and the supernatant was evaporated to dryness using a SpeedVac™ concentrator. Of note, 10 μl of a mixture of methyl esters of fatty acids (FAME, all components purchased from Sigma-Aldrich) were used as markers of the retention index, as previously described (Fiehn, 2016). Moreover, 10 μl of methoxyamine hydrochloride/pyridine (20 mg/ml) (Sigma-Aldrich) were added to dry samples and maintained at 30°C for 90 min on a shaker. Subsequently, 90 μl of N-methyl-N-(trimethylsilyl) trifluoroacetamide were added with 1% trimethylchlorosilane as a derivatizing agent (Sigma-Aldrich), incubated at 37°C for 30 min on a hot plate, and stirred thrice during incubation to ensure complete dissolution. Samples were transferred to a 250-μl glass vial insert in a 1.5-ml glass vial with a screw cap for gas chromatography-mass spectrometry (GC-MS) analysis.

### Gas Chromatography-Mass Spectrometry Untargeted Metabolomics

Derivatized samples were injected in an Agilent 7890B GC system connected to an electron impact (EI) ionization mode 5977A mass selective detector (Agilent Technologies, Palo Alto, CA, USA). A 1 μl aliquot was injected in a splitless injector mode onto a 30-m × 0.25-mm × 0.25-μm DB-5 column (Agilent Technologies, Palo Alto, CA, USA). The injector port temperature was maintained at 250°C, and the helium carrier gas flow rate was set at 1 ml/min at an initial oven temperature of 60°C. The oven temperature was increased at 10°C/min to 325°C for a final run time of 37.5 min. Full-spectra/s (50−600 m/z; 1.7 scans/s) with a digital scan rate of 20 Hz were acquired after a 5.9 min solvent delay, with an MS ion source temperature of 250°C and quadrupole temperature of 150°C. All derivatized samples were run within 24 h after preparation. The mixture of the FAME standard solution C8–C30 was injected to acquire retention times for the calculation of Fiehn’s retention index of the metabolites (Fiehn, 2016). Raw MS data (“.D” file format) were transformed into the ABF format using the Reifycs Abf Converter software (RIKEN Center for Sustainable Resource Science, Yokohama, Japan) prior to data pretreatment. The identification of metabolites was performed as previously described (Fiehn, 2016). Briefly, peak detection, deconvolution, and peak alignment in data processing were performed using the MS-DIAL 2.83 software (Tsugawa et al., 2015) to process the total ion chromatogram and the EI–MS spectra of each GC peak. After deconvolution, the purified mass spectrum of each of the trimethylsilylated metabolites was identified, and deconvoluted peaks were matched against the Fiehn’s mass spectral library. Library match hits were ranked against experimental data based on the total retention index and mass spectral similarity across all samples processed in a batch. The retention index Fiehn RI—based on FAME—was used. Metabolites were identified by matching the EI–MS spectra with those of reference compounds from Fiehn’s mass spectral library. For the analysis, we used a 2,000 retention index tolerance, 70% EI similarity cut-off, 70% identification score cut-off, 0.5 Da m/z tolerance, and 0.5 min retention time tolerance. Data were tabulated in a Microsoft Excel (Microsoft, Redmond, WA, USA) spreadsheet (“.csv” file format).

### Gas Chromatography-Mass Spectrometry Data Analyses

Statistical analysis and metabolic mapping were performed using the MetaboAnalyst 4.0 on-line platform (https://www.metaboanalyst.ca/) (Chong et al., 2018). The “.csv” file extracted from the MS-DIAL software was uploaded and data were normalized using the control condition and Pareto scaling. The results obtained were expressed in a logarithmic scale for graphical representation. The metabolic impact was determined by matching the GC-MS data with the Kyoto Encyclopedia of Genes and Genomes (KEGG) pathway database (https://www.genome.jp/kegg/pathway.html) (Kanehisa and Goto, 2000), integrated with the MetaboAnalyst platform (McGill University, Montreal, QC, Canada).

### Evaluation of Cell Viability

NSCLC cells were seeded in 96-well plates at a density of 2.5 × 10^3^ (for H1299 cells) or 5 × 10^3^ (for A549 cells) cells per well. After 24 h, cells were exposed to the drugs for 96 h. Subsequently, cell viability was evaluated through incubation with 3-(4,5-dimethylthiazol-2-yl)-2,5-diphenyltetrazolium bromide (MTT) (0.5 mg/ml, Sigma-Aldrich) for 4 h. The formed crystals were solubilized through incubation with 100 µl of 10% SDS in 0.01 M hydrochloride overnight at 37°C. The production of formazan was measured at 579–690 nm in a Varioskan™ multimode microplate reader (Thermo Fisher Scientific, Waltham, MA, USA) (Tada et al., 1986).

### Drug Combination Studies

Experiments of indomethacin combined with polyamine synthesis inhibitors (i.e., DFMO, SAM486, and MDL72527) were conducted to evaluate their synergistic effects, according to the Loewe model. Viability was evaluated at 96 h through MTT reduction and data were analyzed using the Combenefit software (Di Veroli et al., 2016).

### Statistical Analyses

For the western blotting and qRT-PCR studies, data were analyzed using the GraphPad Prism software (V 7.0; GraphPad Software, San Diego, CA, USA). One-way analysis of variance was performed to compare groups. Dunnett’s post-test was used to compare data with the control group, while Tukey’s post-test was used to perform comparisons between all experimental groups. Metabolomics were evaluated using Student’s t-test. The combination studies were evaluated using the t-test to compare differences between the Loewe’s theoretical model and the obtained data. For all comparisons, a *p* < 0.05 denoted statistical significance.

## Results

### Indomethacin Induced *SAT1* Expression, Increasing the Levels of Spermidine/Spermine-N^1^-Acetyltransferase in Non-Small Cell Lung Cancer Cell Lines

**Figure 1:**
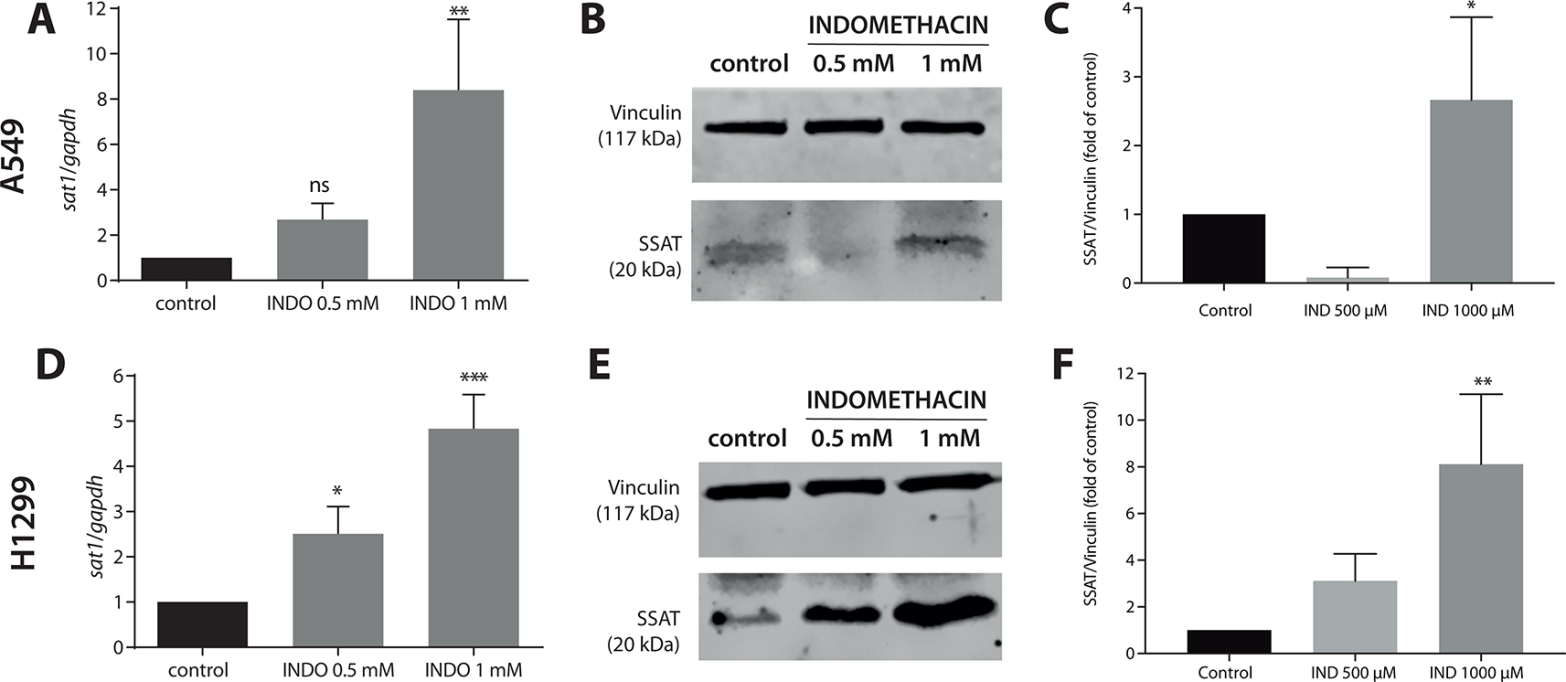
Effect of indomethacin on the expression of *SAT1* and levels of spermidine/spermine-N^1^-acetyltransferase (SSAT) in non-small cell lung cancer (NSCLC) cells. A549 and H1299 cells were exposed to indomethacin 0.5 and 1 mM for 24 h. The messenger RNA (mRNA) levels of *SAT1* and protein levels of SSAT were evaluated using quantitative PCR (qPCR) and western blotting, respectively. **(A)** The mRNA levels of *SAT1* in A549 cells. **(B)** Representative blot of the SSAT protein levels in A549 cells exposed to indomethacin. **(C)** Quantitation of the SSAT levels in A549 cells exposed to indomethacin. **(D)** The mRNA levels of *SAT1* in H1299 cells. **(E)** Representative blot of the SSAT levels in H1299 cells exposed to indomethacin. **(F)** Quantitation of the SSAT levels in H1299 cells exposed to indomethacin. *p < 0.05; **p < 0.01, ***p < 0.0001 and ns, no significant compared with untreated control, calculated using one-way ANOVA and Dunnett’s post-test. Data summarize the results of three independent experiments.

In A549 cells, treatment with indomethacin 1 mM resulted in an 8.4 ± 3.1-fold increase in the expression of *SAT1 versus* control cells (*p* < 0.001) (**Figure 1**, and **Supplementary Figure S2**). In H1299 cells, indomethacin induced a 2.5 ± 0.6-fold (0.5 mM) and 4.8 ± 0.8-fold (1 mM) (*p* < 0.005 and *p* < 0.0001, respectively) increase in the expression of *SAT1*. The evaluation of SSAT protein levels revealed a concentration-dependent increase in A549 and H1299 cells. The difference *versus* control cells was significant at indomethacin 1 mM for both cell lines [2.7-fold increase for A549 cells (*p* = 0.047) and 8.11-fold increase for H1299 cells (*p* = 0.0061)] (**Figures 1B, C, E**).

### Indomethacin Impaired Polyamine and Amino Acid Metabolism in Non-Small Cell Lung Cancer Cells

**Figure 2:**
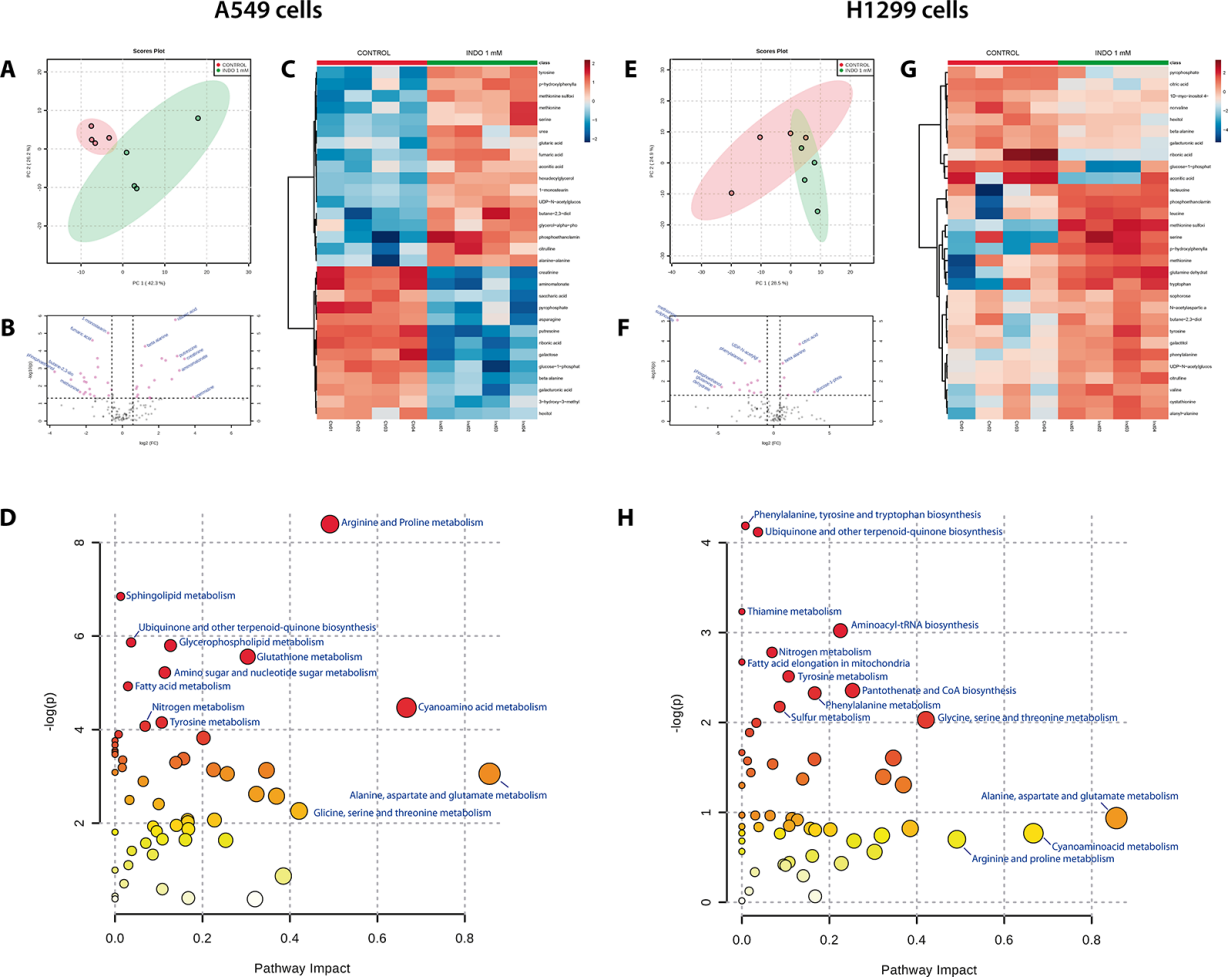
Metabolomic analyses of non-small cell lung cancer (NSCLC) cells exposed to indomethacin. A549 and H1299 cells were exposed to indomethacin for 24 h. An unbiased metabolomics analysis was performed using gas chromatography/mass spectrometry (GC/MS). Identified compounds were analyzed using the MetaboAnalyst platform. **(A, E)** Principal component analyses (PCA) plots of A549 **(A)** and H1299 **(E)** cells. **(B, F)** Volcano plots showing metabolites identified in A549 **(B)** and H1299 **(F)** cells. Fold-change and *p* value thresholds were set at 1.5 and 0.05, respectively. The notated compounds in each graph are those which demonstrated statistical significance compared with untreated cells. **(C, G)** Heatmaps showing the 30 metabolites with greater variation observed in A549 **(C)** and H1299 **(G)** cells, comparing treated and untreated cells. **(D, H)** Overview of the pathway analysis in A549 **(D)** and H1299 **(H)** cells exposed to indomethacin. The scatter plots represent the pathway impact value and *p* value from the pathway topology analysis of the differentially expressed metabolite. The position of each node in the plot is based on its pathway impact (X-axis) value and *p* value (Y-axis). Pathways which demonstrated statistical significance (*p* < 0.05, compared with untreated cells) are shown in red. Data summarize the results of four independent experiments.

After GC-MS data matching with Fiehn’s GC-MS library, 134 metabolites were identified. The results showed that indomethacin exerted different metabolic effects on the two NSCLC lines investigated (**Figure 2**). In A549 cells, indomethacin induced a clear pattern of metabolic alteration (**Figure 2A**), resulting in significant changes in 11 metabolic features (**Figure 2B**) and >1.5-fold changes in >30 metabolic features compared with control cells (**Figure 2C**). On the other hand, in H1299 cells, indomethacin resulted in a lower metabolic impact, with significant changes observed in only eight features (**Figure 2F**) and a diffuse pattern of changes in the identified metabolites (**Figures 2E, G**).

Matching of the metabolomic data with the KEGG pathway database (Kanehisa and Goto, 2000; Chong et al., 2018) showed that, in both types of cells, the most greatly impacted pathways (in terms of the number of metabolites with variation inside the pathway) were those involving the metabolism of alanine, aspartate, glutamate, arginine, proline, and cyanoamino acids (**Figures 2D, H**, and **Table 1**). However, the variation in these pathways was statistically significant only in A549 cells (**Table 1**).

**Table 1:** Pathway analysis of non-small cell lung cancer (NSCLC) cells exposed to indomethacin.

			A549 cells		H1299 cells	
KEGG pathway	Match status	Impact	*p*	FDR	*p*	FDR
Alanine, aspartate, and glutamate	24/11	0.860	0.047	0.100	0.392	0.647
Cyanoamino acid metabolism	16/5	0.670	0.011	0.079	0.464	0.647
Arginine and proline metabolism	77/14	0.490	0.00002	0.013	0.496	0.647
Glycine, serine, and threonine metabolism	48/10	0.420	0.104	0.179	0.131	0.623
Taurine and hypotaurine metabolism	20/4	0.380	0.419	0.471	0.440	0.647
Citrate cycle (TCA cycle)	20/7	0.370	0.076	0.144	0.271	0.647
Cysteine and methionine metabolism	56/9	0.350	0.044	0.100	0.201	0.647
Beta-alanine metabolism	28/7	0.320	0.073	0.143	0.248	0.647
Pyruvate metabolism	32/3	0.320	0.686	0.686	0.476	0.647
Glutathione metabolism	38/9	0.300	0.004	0.042	0.571	0.697
Starch and sucrose metabolism	50/5	0.260	0.047	0.100	0.505	0.647
Pantothenate and CoA biosynthesis	27/7	0.250	0.195	0.249	0.095	0.597
Aminoacyl-tRNA biosynthesis	75/19	0.230	0.043	0.100	0.049	0.597
Lysine degradation	47/4	0.230	0.127	0.205	0.649	0.732
Pyrimidine metabolism	60/8	0.200	0.022	0.098	0.445	0.647
Galactose metabolism	41/6	0.170	0.126	0.205	0.204	0.647
Phenylalanine metabolism	45/8	0.170	0.132	0.208	0.098	0.597
Lysine biosynthesis	32/4	0.170	0.153	0.222	0.447	0.647
Inositol phosphate metabolism	39/2	0.170	0.671	0.686	0.936	0.953
Purine metabolism	92/9	0.160	0.034	0.100	0.440	0.647

A549 and H1299 cells were exposed to indomethacin 1 mM for 24 h. Metabolites were measured using GC-MS and the metabolomic analyses were performed using the MetaboAnalyst 4.0 platform matching data with the KEGG pathway database. “Match status” shows the matched number of compounds in the data, related to the total compounds in the pathway. “Impact” is the pathway impact value calculated from the pathway topology analysis. The *p* value was calculated from the enrichment analysis comparing the treated *versus* untreated cells and was further adjusted using the false discovery rate method (FDR column).

**Figure 3:**
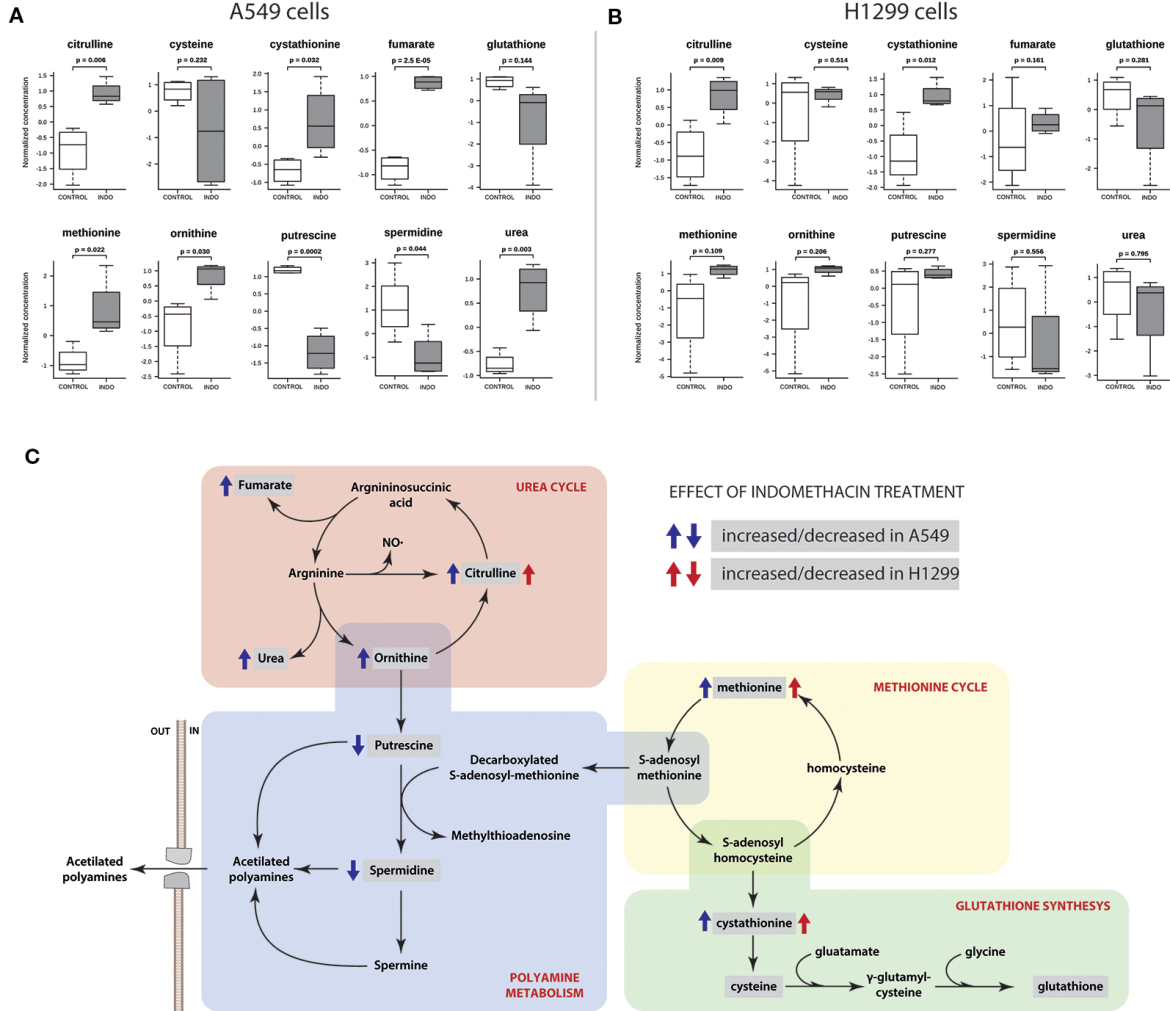
Polyamine pathway-related metabolite profiling of non-small cell lung cancer (NSCLC) cells exposed to indomethacin. A549 and H1299 cells were exposed to indomethacin for 24 h. An unbiased metabolomics analysis was performed using gas chromatography/mass spectrometry (GC/MS). Identified compounds were analyzed using the MetaboAnalyst platform. **(A, B)** Analyses of 10 selected metabolites after treatment with indomethacin in A549 **(A)** and H1299 **(B)** cells. The *p* values were calculated using the Student’s t-test between treated and untreated cells. **(C)** Pathway mapping showing the polyamine metabolism and related pathways. Identified metabolites are enclosed in gray boxes. Blue and red arrows indicate the change (i.e., increase or decrease) of each metabolite in A549 or H1299 cells after exposure to indomethacin. Data summarize the results of four independent experiments.

The pathway limits are arbitrary, and the pathway of polyamine metabolism is not defined in the KEGG database. Therefore, we observed in detail the metabolomic data regarding the metabolic neighborhood of polyamine metabolism. Specific changes in the two detected polyamines (i.e., putrescine and spermidine) and the closely related metabolic pathways (i.e., arginine metabolism, methionine cycle, and glutathione synthesis) were evaluated. As shown in **Figure 3A**, the levels of putrescine and spermidine were significantly decreased in A549 cells after exposure to indomethacin (*p* = 0.0002 and *p* = 0.044, respectively, *versus* control cells). However, in H1299 cells, the observed changes were not statistically significant (**Figure 3B**). Moreover, A549 cells exhibited a significant increase upstream of the putrescine synthesis pathway, with a significant increase in the levels of ornithine (*p* = 0.03). Interestingly, the levels of ornithine-related metabolites (i.e., citrulline, urea, and fumarate) were also increased. These metabolites are involved in the urea cycle, suggesting that this metabolic pathway may be activated in lung cancer cells exposed to indomethacin (**Figure 3A**). Furthermore, in A549 cells, there was a significant increase (*p* = 0.022) in the levels of methionine—the precursor of S-adenosyl-methionine. The latter is in turn decarboxylated by AMD1, one of the rate-limiting steps in the synthesis of spermidine (**Figure 3C**) (Casero et al., 2018). However, these metabolic changes were not observed in H1299 cells and the levels of polyamines remained unaltered.

### Polyamine Synthesis Enzymes Were Differentially Expressed in Non-Small Cell Lung Cancer Cell Lines

**Figure 4:**
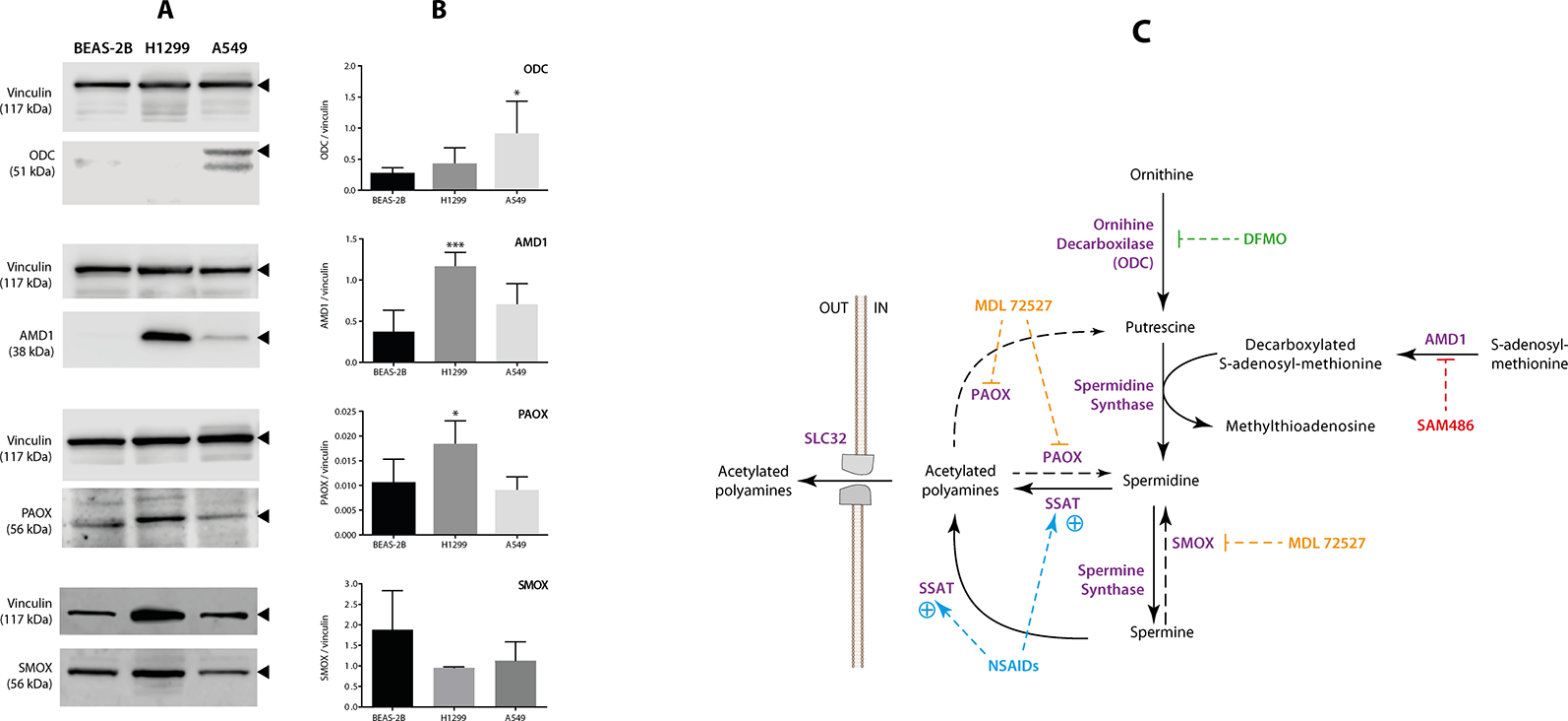
Measurement of the levels of polyamine-related enzymes in non-small cell lung cancer (NSCLC) cells. The levels of four polyamine-related enzymes: ornithine decarboxylase (ODC), adenosyl-methionine decarboxylase (AMD1), polyamine-oxidase (PAOX), and spermine oxidase (SMOX) were evaluated using western blotting in two NSCLC cell lines (A549 and H1299) and one normal epithelial lung cell line (BEAS-2B). The levels of vinculin were used as internal control. **(A)** Representative blots of vinculin, ODC, AMD1, PAOX, and SMOX in the BEAS-2B, A549, and H1299 cells. **(B)** Quantitation of ODC, AMD1, PAOX, and SMOX levels in the three cell lines. Protein levels are expressed as the ratio of protein/vinculin in each sample. The graph summarizes five independent measurements. *p < 0.05 and ***p < 0.001, compared with BEAS-2B cells, calculated using one-way ANOVA. **(C)** Schematic view showing the role of each polyamine pathway enzyme. The panel also shows the best-known inhibitors of ODC [eflornithine, (DFMO)], AMD1 (SAM486), and PAOX/SMOX (MDL72527).

As shown in **Figures 4A, B**, and **Supplementary Figure S3** both NSCLC cell lines exhibited a differential profile in terms of the levels of polyamine synthesis enzymes compared with BEAS-2B. Although the levels of ODC were higher in A549 cells (*p* < 0.05, compared with control cells), those of PAOX and AMD1 were higher in H1299 cells (*p* < 0.05 and *p* < 0.001, respectively, compared with control cells). Finally, the levels of SMOX were not different between cells lines. These findings suggest that, unlike in A549 cells, acetylated polyamines can be effectively “recovered” in H1299. A schematic view of the enzymes involved in the synthesis of polyamines is shown in **Figure 4C**.

### Indomethacin Exerted a Synergistic Effect in Combination With Adenosyl-Methionine Decarboxylase and Polyamine-Oxidase/Spermine Oxidase Inhibitors

**Figure 5:**
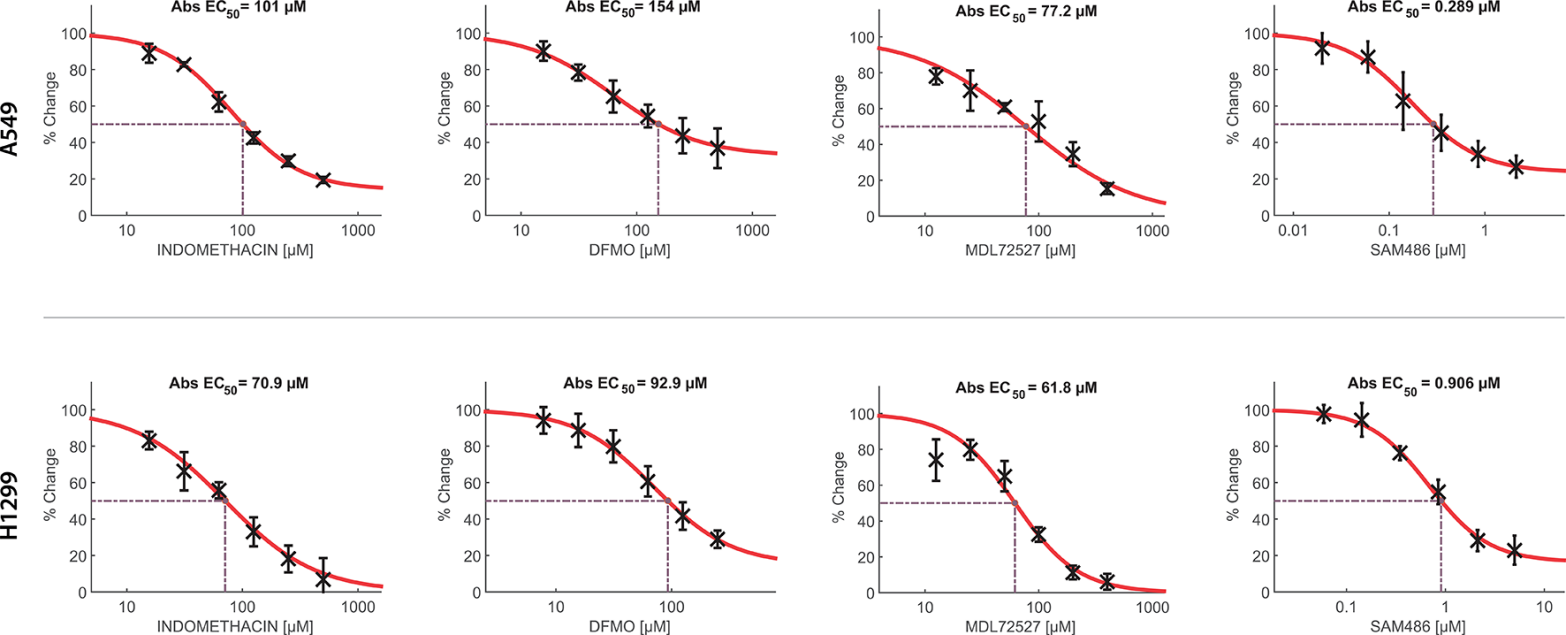
Concentration-response curves of indomethacin, eflornithine (DFMO), MDL72527, and SAM486 for the viability of non-small cell lung cancer (NSCLC) cells. The effects of indomethacin, DFMO, MDL72527, and SAM486 on cell viability were measured using the 3-(4,5-dimethylthiazol-2-yl)-2,5-diphenyltetrazolium bromide (MTT) assay. Two NSCLC cell lines were used: A549 (upper row) and H1299 (lower row). Cells were exposed to different drugs for 96 h. Data were loaded in the Combenefit software to plot concentration-response curves and combination studies (**Figure 6**). “Abs EC_50_” indicates the concentration that reduced cell viability by 50%, normalized using control data. Data summarize the results of four independent experiments.

Furthermore, we evaluated the effect of indomethacin combined with inhibitors of the key enzymes of the polyamine synthesis: DFMO, inhibitor of ODC; SAM486A, inhibitor of AMD1; and MDL72527, a pan-oxidase inhibitor that can inhibit PAOX and SMOX enzymes (**Figure 4C**). When each inhibitor was assayed alone, the sensitivity of each cell line correlated with the relative level of each polyamine synthesis enzyme (**Figure 5**). Thus, A549 cells—which overexpress the ODC enzyme—were 1.65-fold more resistant to DFMO than H1299 cells [half maximal effective concentration (EC_50_) = 154 *versus* 92.9 µM, respectively]. In contrast, H1299 cells—which overexpress the AMD1 enzyme—were 3.13-fold more resistant to the effect of SAM486 (i.e., the AMD1 inhibitor) (EC_50_ = 154 *versus* 92.9 µM, respectively). Regarding MDL7252—the PAOX/SMOX inhibitor—the EC_50_ was 77.2 *versus* 61.6 µM, respectively, representing an increase of 1.25-fold. Despite the overexpression of PAOX in H1299 cells, we hypothesized that the activity of PAOX becomes important following an increase in the activity of SSAT.

**Figure 6:**
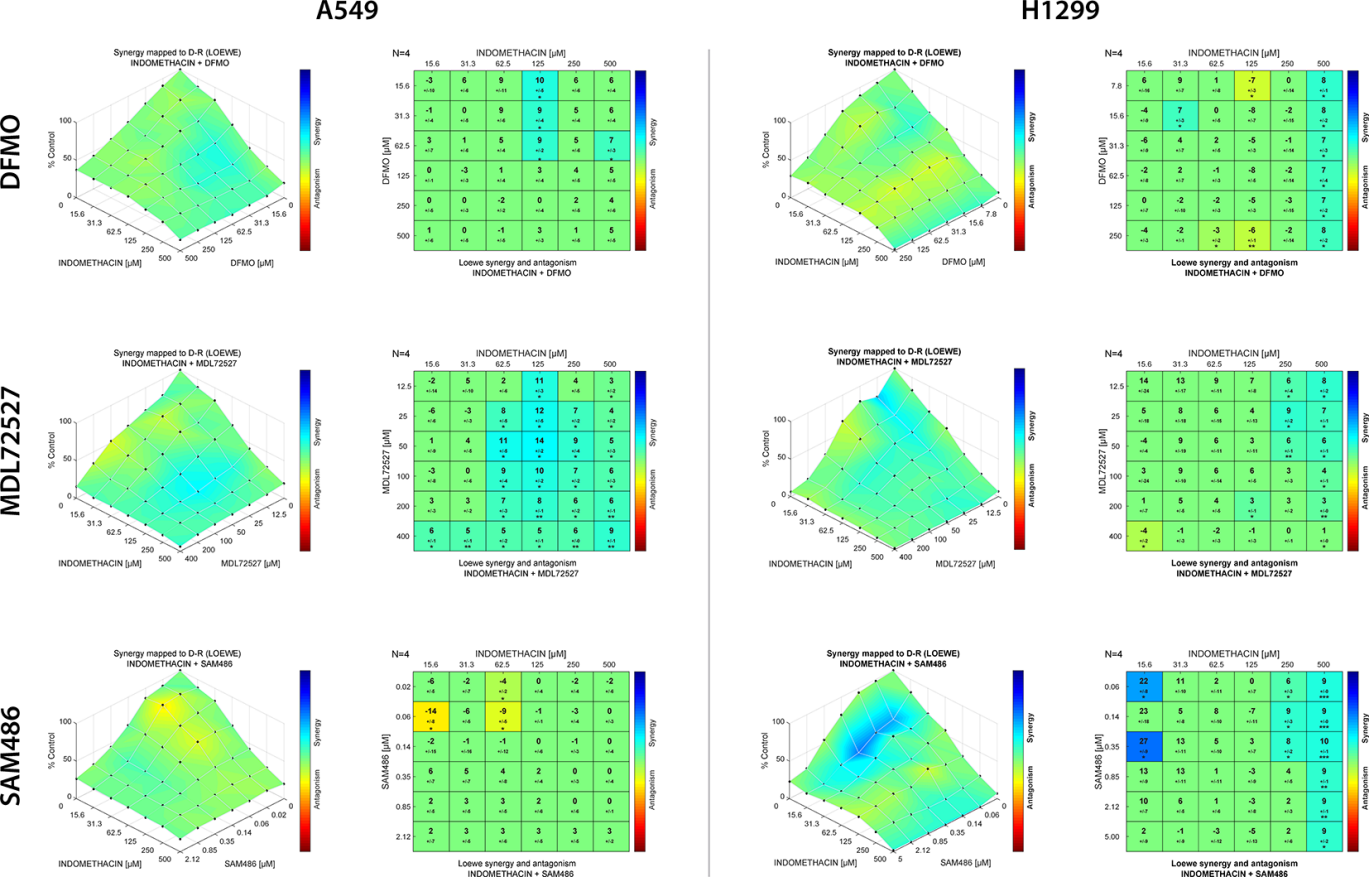
Effect of indomethacin combined with eflornithine (DFMO), SAM486A, and MDL72527 on the viability of non-small cell lung cancer (NSCLC) cells. The effects of the combination of indomethacin with DFMO (upper row), MDL72527 (middle row), and SAM486A (lower row) on cell viability were measured using the 3-(4,5-dimethylthiazol-2-yl)-2,5-diphenyltetrazolium bromide (MTT) assay in two NSCLC cell lines: A549 (left panels) and H1299 (right panels). Cells were exposed to the different drugs for 96 h. For the drug combinations, two-fold dilution series comprising six concentrations, were mixed in every possible combination. Effects matrices were plotted using the Combenefit software. This software constructs a XYZ model of the combination, using the effect of each drug alone and Loewe’s model of drug additivity. Differences between the theoretical combinations and empirical data are represented by a number generated for each combination point. Positive numbers represent synergistic combinations, whereas negative numbers indicate antagonistic interactions. The color code shows combination points that are significantly different from the theoretical model, calculated using Student’s t-test. *p < 0.05, **p < 0.01, and ***p < 0.001. The graphs show the mean ± standard deviation of four independent experiments.

Finally, the synergistic effect of indomethacin with different polyamine synthesis inhibitors was evaluated using Loewe’s synergy model. As shown in **Figure 6**, DFMO exhibited a weak synergistic effect when combined with indomethacin in A549 cells. However, this effect was significant in H1299 cells at the highest concentration of indomethacin. Interestingly, MDL72527 combined with indomethacin at concentrations exceeding 62.5 and 25 µM, respectively, showed significant synergy in A459 cells. In H1299 cells, this combination exhibited synergy at concentrations of indomethacin >250 µM. When the combination of SAM486 and indomethacin was investigated, there was no synergy observed in A549 cells. However, two synergy spots were observed at 15.6 µM and at the highest concentrations of indomethacin (250 and 500 µM).

## Discussion

The main mode of action described for NSAIDs is the inhibition of the cyclooxygenase enzyme, which is involved in the inflammatory cascade. However, the overexpression of SSAT is described as a cyclooxygenase-independent effect, as shown for sulindac, acetylsalicylic acid, celecoxib, ibuprofen, and naproxen in several models of cancer (Babbar et al., 2003; Babbar et al., 2006a; Wood et al., 2011; Hughes et al., 2012; Xie et al., 2012). In this study, we demonstrated that indomethacin induced an increase in SSAT expression, with a metabolic impact on NSCLC cells.

The molecular mechanism underlying the effects of NSAIDs on SSAT expression appears to differ among NSAIDs. For instance, it has been described that sulindac is able to stimulate the peroxisome proliferator-activated receptor γ (PPARγ), inducing its binding to the PPAR-response elements present in the *SAT1* gene (Babbar et al., 2003). On the other hand, the effect of acetylsalicylic acid is mediated by activation of the nuclear factor κB (NF-κB) pathway (Babbar et al., 2006a). The latter mechanism has also been associated with tumor necrosis factor α-induced SSAT expression in NSCLC cells (Babbar et al., 2006b). In this setting, it is probable that the effect of indomethacin is mediated by PPARγ, owing to the observation that indomethacin binds and activates PPARγ (Lehmann et al., 1997). However, its effect on the nuclear factor-κB pathway has only been suggested through indirect measurements (Carrasco-Pozo et al., 2016).

As expected, exposure to indomethacin led to a statistically significant decrease in the levels of spermidine in A549 cells. However, the polyamine content of H1299 cells was not modified (**Figure 3**). A previous study involving a model of transfection-induced overexpression of SSAT showed an increase in the levels of putrescine, presumably through the activity of the PAOX enzyme (Mandal et al., 2013). Surprisingly, in our model, the levels of putrescine were decreased in A549 cells exposed to indomethacin (**Figure 3**). The depletion of putrescine after treatment with indomethacin has been previously reported in colon cancer cells and related to a decrease in ODC activity (Turchanowa et al., 2001). The present findings in A549 cells overexpressing *ODC* are consistent with these previous data (**Figure 4**).

In our model, one of the most important differences is the presence of a mutation in the Kirsten rat sarcoma viral oncogene homolog (KRAS) gene (codon 12) in A549 cells, but not in H1299 cells. Mutations in *KRAS* are present in approximately 25% of patients with NSCLC. This is the most common gain-of-function genetic alteration observed in NSCLC patients in Western countries. *KRAS* encodes a small GTPase of the Ras superfamily, which activates several pathways related to cell survival and proliferation [e.g., the rapidly accelerated fibrosarcoma (RAF)/mitogen-activated protein kinase kinase (MEK)/extracellular signal-regulated kinase (ERK) and phosphoinositide 3-kinase (PI3K)/AKT/mammalian target of rapamycin (mTOR) pathways] (Ferrer et al., 2018). An increase in the levels of ODC in *KRAS*-mutated compared with *KRAS* wild-type NSCLC or normal lung cells (**Figure 4**) has not been previously reported. This may be attributed to *KRAS* mutations closely related to the activation of the *c-Myc* oncogene (Ferrer et al., 2018), which exerts a direct effect on the *ODC* promoter (Bello-Fernandez et al., 1993). Accordingly, the use of antisense oligonucleotides against *c-Myc*, inhibits the expression of *ODC* and lung tumorigenesis *in vivo* (Yano et al., 2001).

In addition, a “correction” of *ODC* overexpression *via* treatment with indomethacin in colon cancer cells (HCT-116), which carry a mutation in the KRAS gene, was previously reported (Turchanowa et al., 2001). Thus, similar phenomena may also contribute to the greater impact that this drug exerts on the levels of putrescine in A549 cells (**Figure 3**).

The metabolomic analyses revealed that the levels of metabolites related to the urea cycle were increased in A549 cells after exposure to indomethacin. The enzymes involved in the complete urea cycle are typically expressed in hepatocytes. However, it has been described that cancer cells induce the urea cycle to support the supply of intermediates required for essential biochemical processes, such as the polyamine pathway, tricarboxylic-acid cycle, and synthesis of pyrimidine (Keshet et al., 2018). It is important to note that a differential pattern of urea cycle metabolism has been observed between NSCLC cell lines, mainly related to the presence of mutations in the *KRAS* gene. In cells with a *KRAS* mutation (e.g., A549), a decrease in the expression of the liver kinase B1 has been observed, allowing the overexpression of the carbamoyl phosphate synthetase-1 and synthesis of carbamoyl phosphate, which is in turn used for the synthesis of citrulline from ornithine (Kim et al., 2017). On the other hand, H1299 cells—containing the wild-type *KRAS* gene—do not overexpress carbamoyl phosphate synthetase-1, without induction of the urea cycle (Celiktas et al., 2017). However, in a model of H1299 cells*-*mutated *KRAS*, the urea cycle and polyamine metabolism are upregulated with a significant increase in the levels of putrescine and spermidine (Caiola et al., 2018).

Indomethacin is currently considered a first-line drug for inducing the closure of the ductus arteriosus in preterm infants, with an acceptable safety profile (Marconi et al., 2019). Also, indomethacin was markedly effective in other clinical settings, such as against renal pain (Pathan et al., 2018) and some types of headache (e.g., hemicrania continua) (Mehta et al., 2018). However, there are few reports indicating its effect in human cancers. In a randomized study of 135 patients with different types of advanced cancer (mainly liver, pancreas, gastric, and colon cancer), indomethacin decreased the inflammatory burden and suffering in patients, and doubled the survival expectancy compared with placebo (Lundholm et al., 1994). More interestingly, an observational retrospective study of NSCLC patients who received indomethacin or ibuprofen for the treatment of pain after cancer surgery found that the NSAID-treated group had significantly longer survival and disease-free progression (Jiang et al., 2018). Although Jiang et al. did not discriminate the results between ibuprofen and indomethacin, these data suggest that indomethacin plays a potential role in avoiding the recurrence of NSCLC after surgery.

Dual-targeted therapy against polyamine metabolism, using NSAIDs (i.e., celecoxib and sulindac) and DFMO, has been previously tested in several clinical trials for the prevention of colorectal cancer. The combination of these agents demonstrated moderate improvement (Thompson et al., 2010; Lynch et al., 2016). The present study is the first to investigate the combination of NSAIDs with AMD1 and PAOX/SMOX inhibitors in cancer. In our model, the combination of indomethacin and DFMO yielded poor synergy scores in both cellular models. Moreover, the combination of indomethacin with SAM486 did not demonstrate significant synergy in A549 cells. However, it showed an interesting spot of synergy, at low concentrations, in H1299 cells (**Figure 6**). Finally, the combination of indomethacin with MDL72527 showed the greatest synergy in A459 cells. The rationale behind this combination relies in an enhanced flux of acetylated polyamine exportation, due to the inability to “rescue” these acetylated polyamines after inhibition of PAOX, and the inability of cells to recover the level of spermidine from spermine after inhibition of SMOX. Thus, the effect of indomethacin, alone and in combination with MDL72527 may offer a new therapeutic tool. Studies involving animal models of the disease are warranted to investigate this approach.

## Data Availability Statement

Metabolomics data have been deposited to the EMBL-EBI MetaboLights database (Haug et al., 2013), with the identifier MTBLS873. The complete dataset can be accessed at https://www.ebi.ac.uk/metabolights/MTBLS873.

## Author Contributions

FL-C, MM-U, JP-L, LA-L, AR-D, and AM-M performed the experiments and analyzed most data. FL-C, RAB, and PA obtained the GC/MS data and performed the metabolomic analyses. RL-M designed the study, analyzed data, obtained the main funding of the study, created the figures, and wrote the manuscript. All authors read and approved the final manuscript.

## Funding

This work was supported by the Consejo Nacional de Ciencia y Tecnología (Chile), through the Fondo Nacional de Desarrollo Cientifico y Tecnologico program (FONDECYT-1160807), and the Fondo de Equipamiento Cientifico y Tecnologico program (FONDEQUIP-EQM130257). The funder had no role in the study design, data collection and analysis, decision to publish, or preparation of the manuscript.

## Conflict of Interest

The authors declare that the research was conducted in the absence of any commercial or financial relationships that could be construed as a potential conflict of interest.
