# Supplementary material for: Searching for Drug Synergy Against Cancer Through Polyamine Metabolism Impairment: Insight Into the Metabolic Effect of Indomethacin on Lung Cancer Cells
Source: Front Pharmacol. 2020 Feb 28;10:1670. doi: 10.3389/fphar.2019.01670 (PMC7093016; doi:10.3389/fphar.2019.01670)
Supplement: Supplementary file 1 [file DataSheet_1.docx]

**Supplementary Information File.**

**Indomethacin impairs polyamine metabolism, and potentiates the effect of polyamine inhibitors in** **lung cancer cells: a *KRAS* mutation-associated feature?**

López-Contreras F^1,2,¶^, Muñoz-Uribe M^1¶^, Pérez-Laines J^1^, Ascencio-Leal L^1^, Rivera-Dictter A^1^, Martin-Martin A^1^, Burgos RA^1^, Alarcon P^1^, López-Muñoz R^1^*.

^1^Instituto de Farmacología y Morfofisiología, Facultad de Ciencias Veterinarias, Universidad Austral de Chile. Valdivia, Chile.

^2^Escuela de Graduados, Facultad de Ciencias Veterinarias, Universidad Austral de Chile. Valdivia, Chile. Valdivia, Chile.

***Corresponding Author:** Rodrigo López-Muñoz. Instituto de Farmacología y Morfofisiología, Facultad de Ciencias Veterinarias, Universidad Austral de Chile. Valdivia, Chile. Email: [rodrigo.lopez@uach.cl](mailto:rodrigo.lopez@uach.cl).

^¶^These authors contributed equally to this work.

**Supplementary Methods.**

**Reagents for HPLC analyses of polyamines.** Bovine fetal serum (Cat # 04-001-1A, US Origin) was obtained from Biological Industries Inc. (Cromwell, CT, USA). Putrescine (Cat # 110-60-1), spermidine (Cat # 124-20-9) and spermine (Cat # 71-44-3) were obtained from Sigma-Aldrich, (St. Louis, MO, USA). Water grade analysis (Cat # 7732-18-5), sodium hydroxide (Cat # 1310-73-2), sodium bicarbonate (Cat # 144-55-8), dansyl chloride (Cat # 605-65-2) and trichloroacetic acid (Cat # 76-03-9) were obtained from MERK Millipore Corporation (Burlington, MA, USA). Acetone (Cat # 67-64-1) was obtained from Avantor Performance Materials, Inc, (Phillipsburg, NJ, USA).

**Sample and standards preparation.** Polyamines (putrescine, spermidine and spermine) were prepared in a water solution in a concentration of 100 mg/mL each. 5% trichloroacetic acid (TCA) was prepared in water for analysis. A calibration curve for each polyamine was prepared using dilutions at 100, 50, 25, 12.5, 6.25 mg/L in 500 µL of a 5% TCA solution in 1.5 mL. For samples and standards, a 10% TCA solution was added in a 1:1 ratio (500 µL of 10% TCA per 500 µL of SFB sample or standard) and kept at 4°C for 5 minutes. The samples were then precipitated at 14,000 *g* for 10 minutes at 4°C. 500 µL were collected and the pH adjusted (>10.0) using 5M sodium hydroxide. Samples and standards were derivatizated by a dansylation reaction. For this purpose, 100 μL of each sample or standard was mixed with 400 μL of 0.25M sodium bicarbonate, 200 μL of 10 mg/mL dansyl chloride and 300 μL of acetone in a 1.5 mL glass vial with a screw cap e incubated at 60°C for 1 hour. The samples were transferred and filtered to a 1.5 mL glass vial with a screw cap for HPLC analysis.

**HPLC separation and detection.** 20 μL of derivatizated samples or standards were analyzed using an Elite LaChrom HPLC kit (VWR-Hitachi, PA, USA). Samples were separated in an InertSustain C18 column (3 μm 15 cm × 4.6 mm, GL Sciencies, Shinjuku, TKY, Japan). As mobile phase, a mixture of 50% acetonitrile, 25% methanol and 25% water were used at an isocratic flow of 1 mL/min. The column thermostat was set at 35°C. Derivatizated polyamines were detected at 254 nm using an Elite LaChrom spectrophotometric detector with diode array (VWR-Hitachi , PA, USA).

**SUPPLEMENTARY RESULTS**

**Fetal bovine serum has no quantifiable levels of polyamines.** Due that the exact polyamine content in FBS is still an unknown matter and could vary depending on the brand or origin of the serum used, we quantified the polyamine content in the SFB serum used in our experiments.

HPLC analyses of putrescine, spermidine and spermine levels in FBS indicated that polyamines are not present in the serum used in our experiments. This finding agrees with the report of Wang et al (Wang et al., 2018) , which indicates that bovine serum has a high content of diamine oxidase, an enzyme that catabolize spermidine and spermine. An old report from Niskanen and Wharton (Niskanen and Wharton, 1987) also suggest that different sera contain different levels of polyamines and FBS have lower putrescine levels when compared with horse sera. Thus, polyamine content in serum does not affect our results.


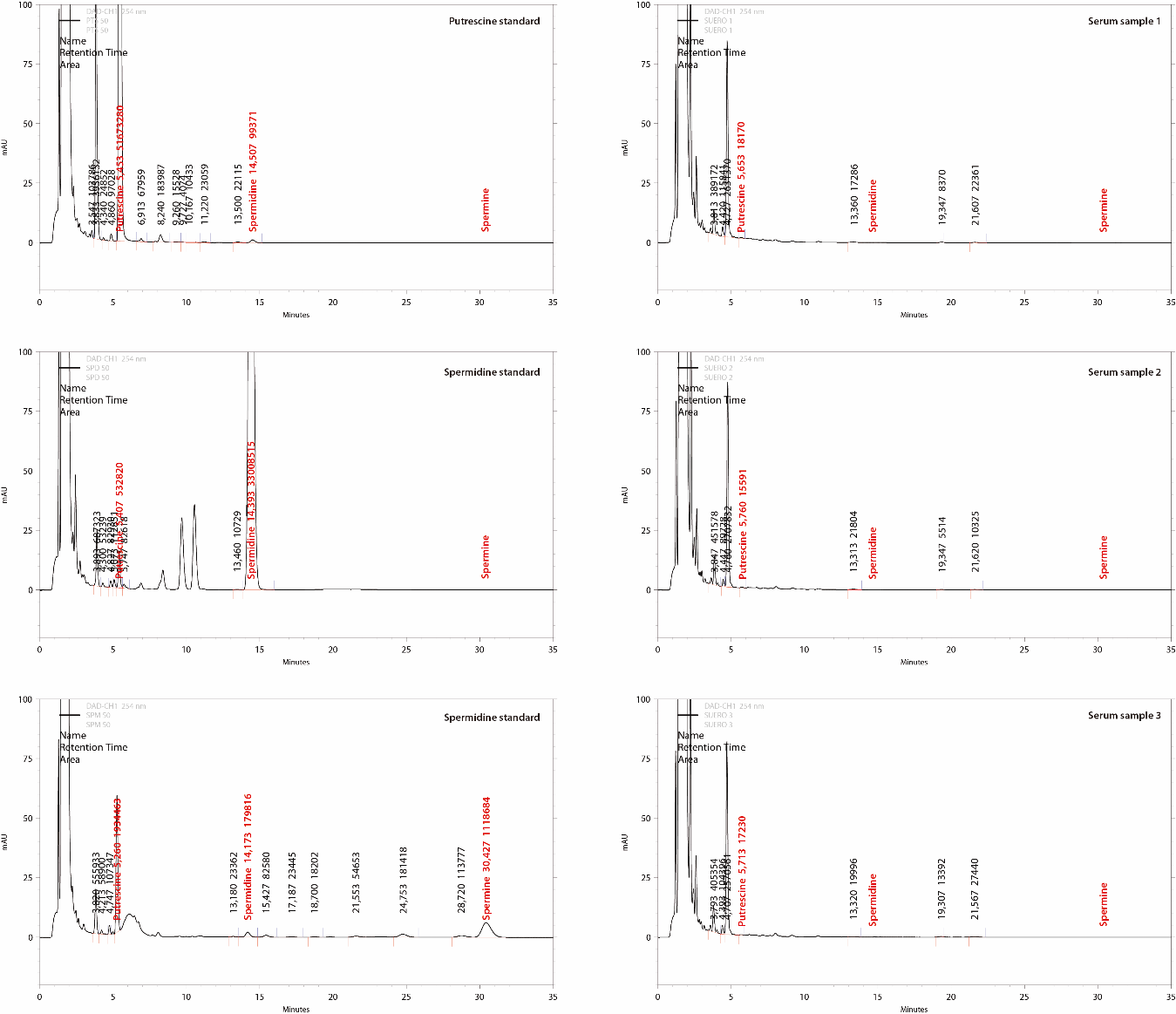


**Supplementary Figure S1. Polyamines content profiling of fetal bovine serum used in this study.** Panels show the chromatographic profile of the three polyamines standards (putrescine, spermidine and spermine, left row) and three samples of fetal bovine serum from the same batch used in our experiments. Over the respective retention time, the polyamine name is indicated in red. The representative chromatograms of each polyamine standard show the standard at 50 mg/L.

**SUPPLEMENTARY FIGURES.**


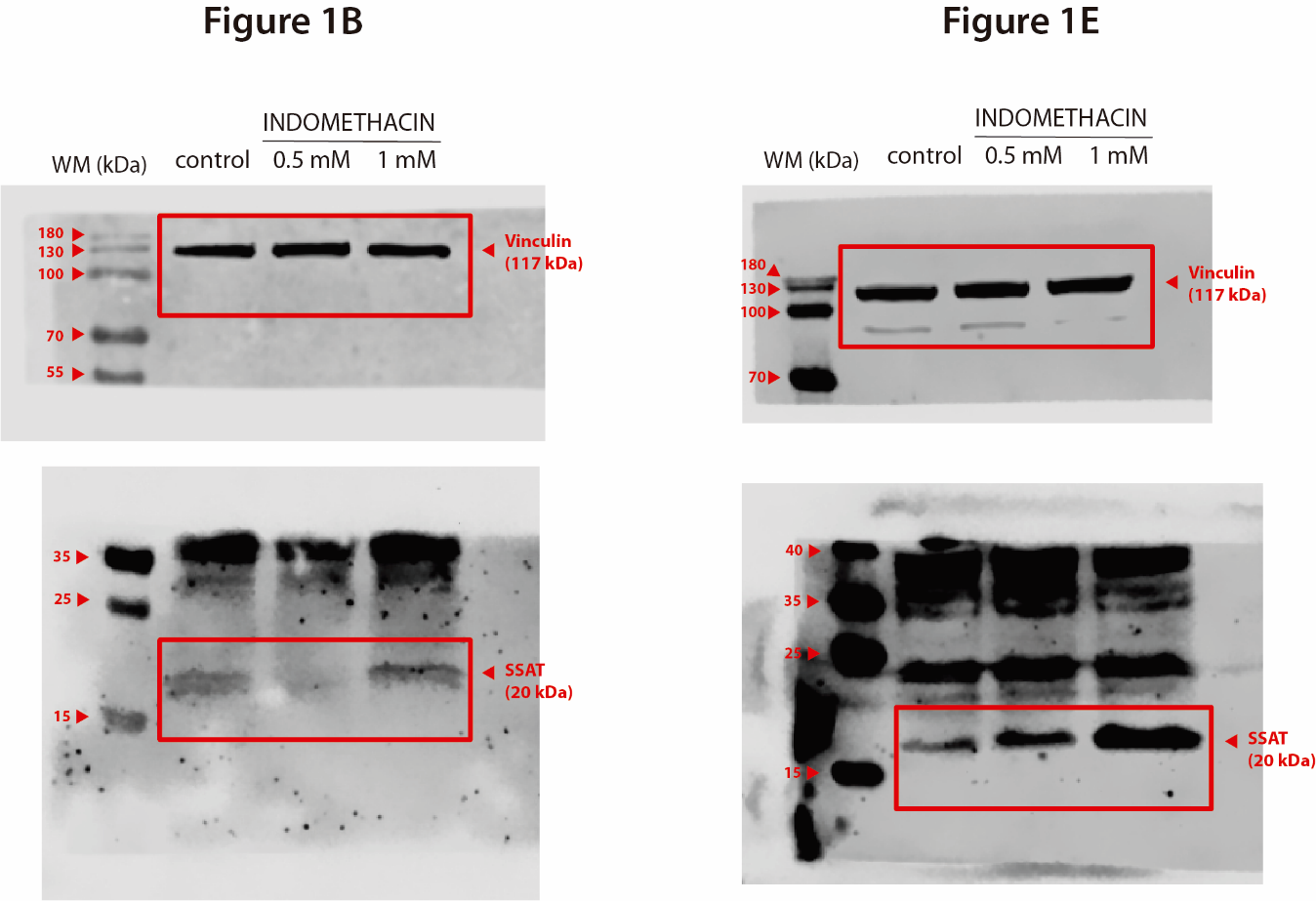


**Supplementary Figure S2. Uncropped blots for images presented in Fig. 1B and 1E.** Red rectangles indicate the cropped section shown in the Fig. 1B and 1E. WM: molecular weight marker. SSAT: spermidine/spermine-N1-acetyltransferase.

**
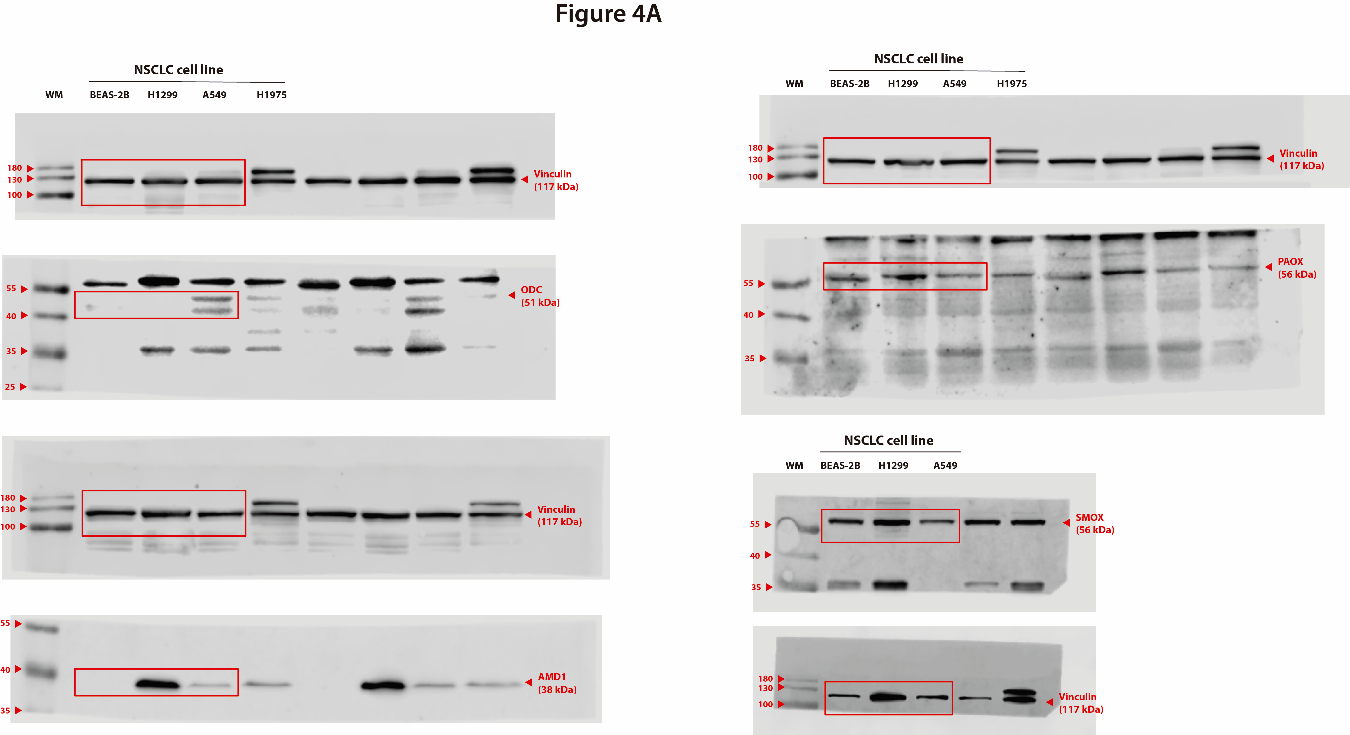
**

**Supplementary Figure S3. Uncropped blots for images presented in Fig. 4A.** Red rectangles indicate the cropped section shown in the Fig. 4A. WM: molecular weight marker. ODC: ornithine decarboxylase, AMD: S-adenosyl-methionine decarboxylase, PAOX: Polyamine oxidase, SMOX: Spermine oxidase

**REFERENCES**

Niskanen, E., and Wharton, W.W., 3rd (1987). Diamine oxidase is important in assessment of polyamine effects on hemopoietic cell proliferation in vitro. *In Vitro Cell Dev Biol* 23**,** 257-260.

Wang, L., Liu, Y., Qi, C., Shen, L., Wang, J., Liu, X., Zhang, N., Bing, T., and Shangguan, D. (2018). Oxidative degradation of polyamines by serum supplement causes cytotoxicity on cultured cells. *Sci Rep* 8**,** 10384.
