# Supplementary figures and images for: Searching for Drug Synergy Against Cancer Through Polyamine Metabolism Impairment: Insight Into the Metabolic Effect of Indomethacin on Lung Cancer Cells
Source: Front Pharmacol. 2020 Feb 28;10:1670. doi: 10.3389/fphar.2019.01670 (PMC7093016; doi:10.3389/fphar.2019.01670)

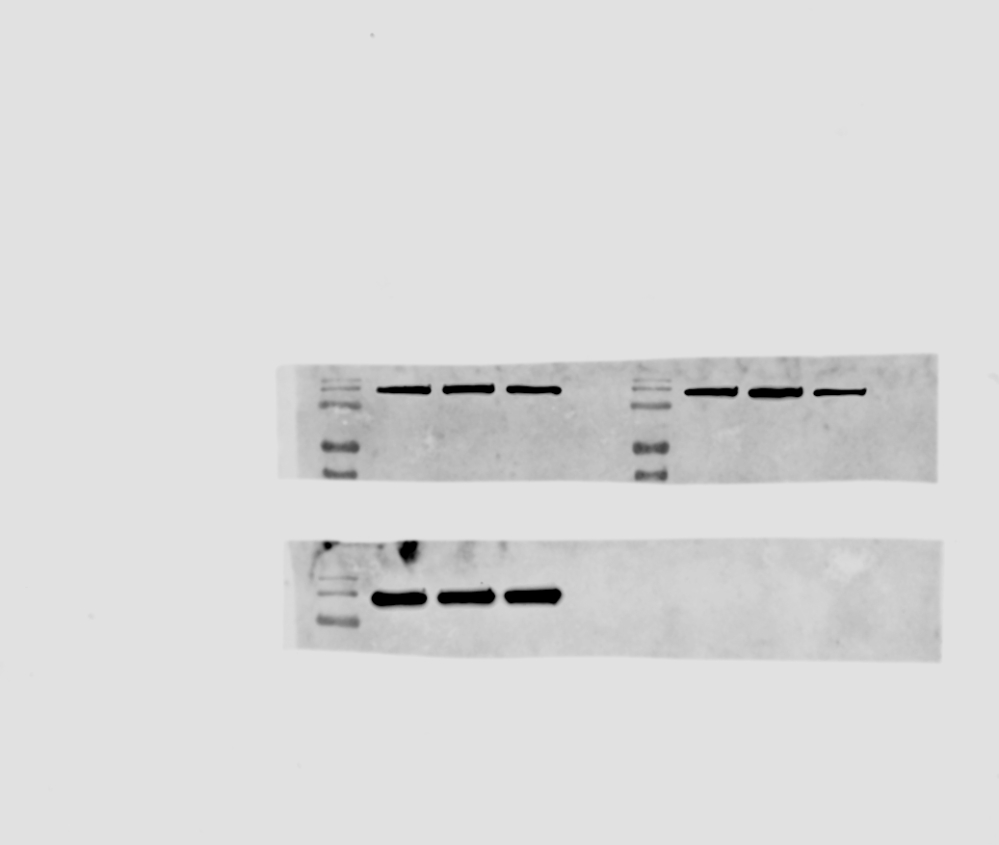

Supplement: Supplementary file 2 [file DataSheet_1.zip › WB original scans/Figure S1 Scan 01.jpg]

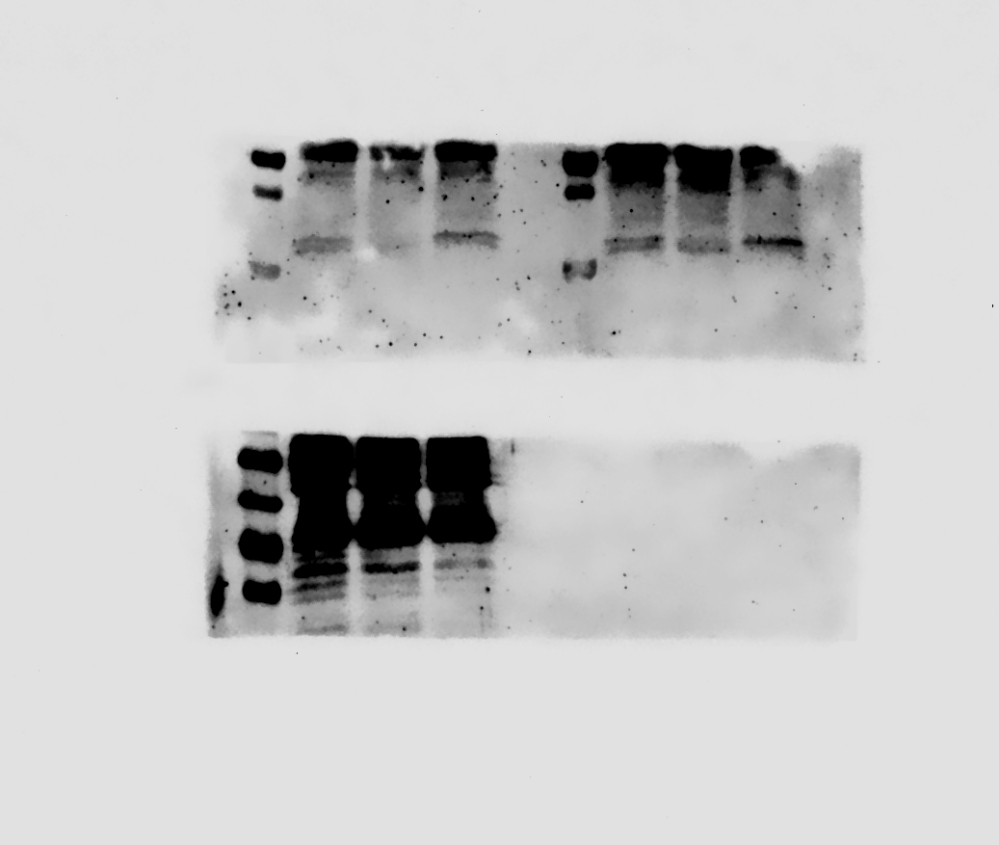

Supplement: Supplementary file 2 [file DataSheet_1.zip › WB original scans/Figure S1 Scan 02.jpg]

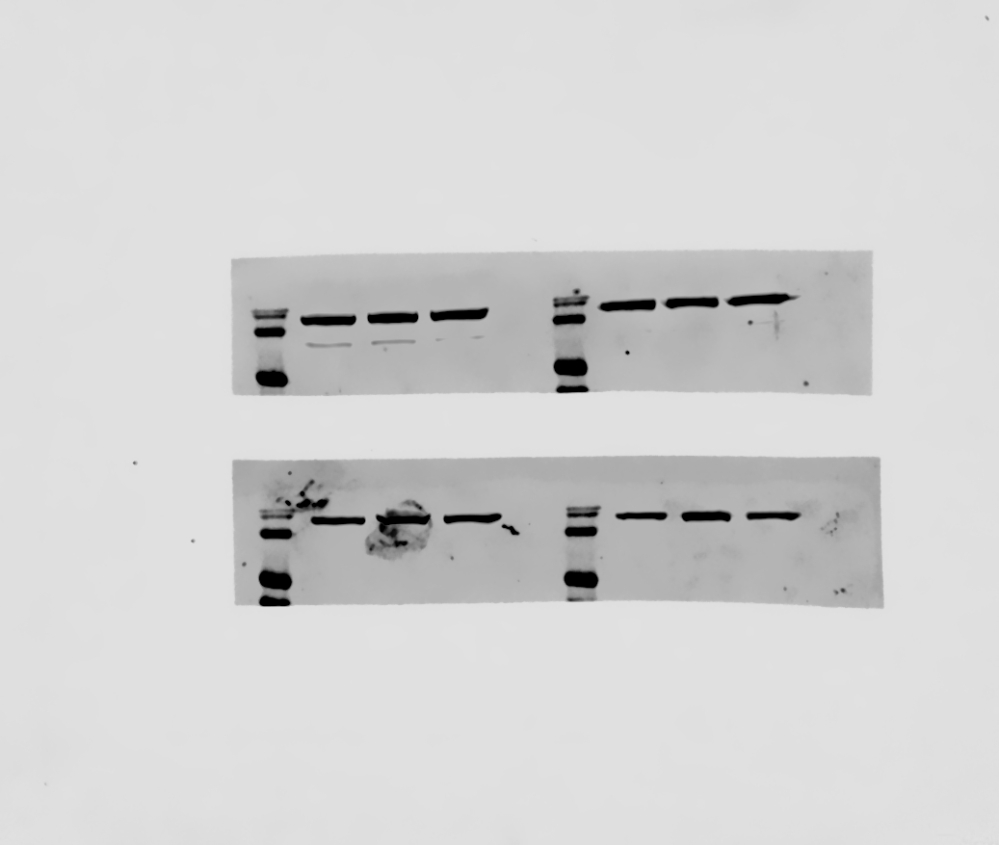

Supplement: Supplementary file 2 [file DataSheet_1.zip › WB original scans/Figure S1 Scan 03.jpg]

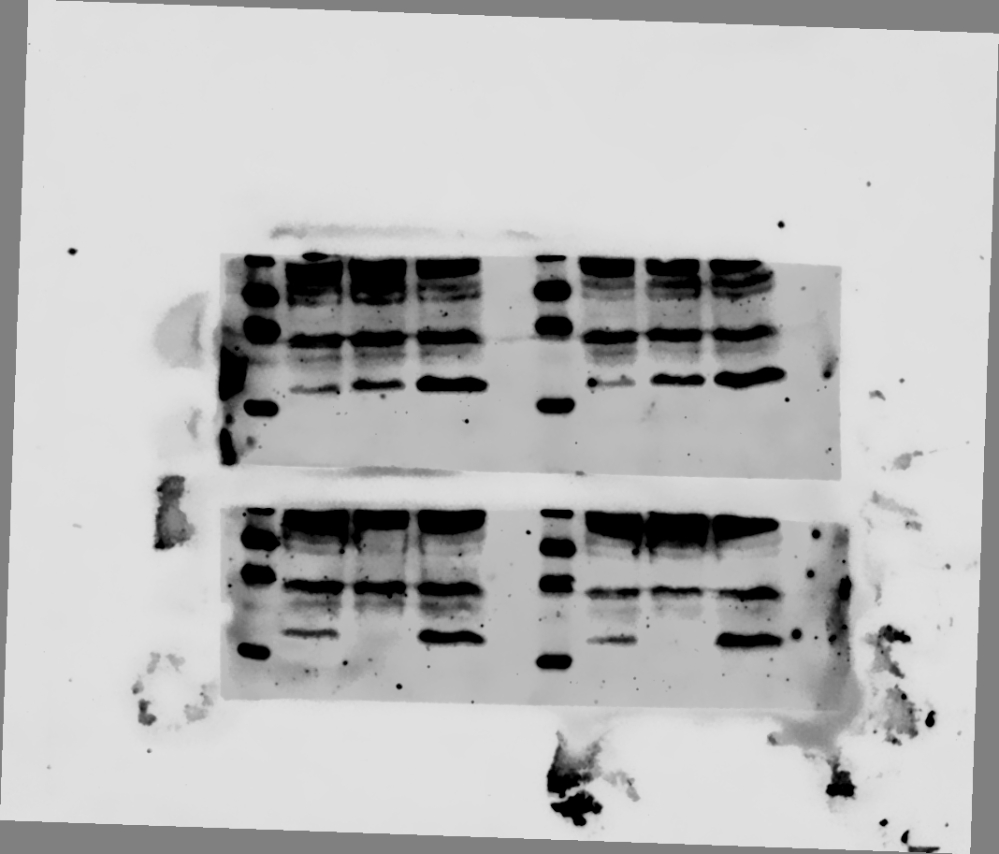

Supplement: Supplementary file 2 [file DataSheet_1.zip › WB original scans/Figure S1 Scan 04.jpg]

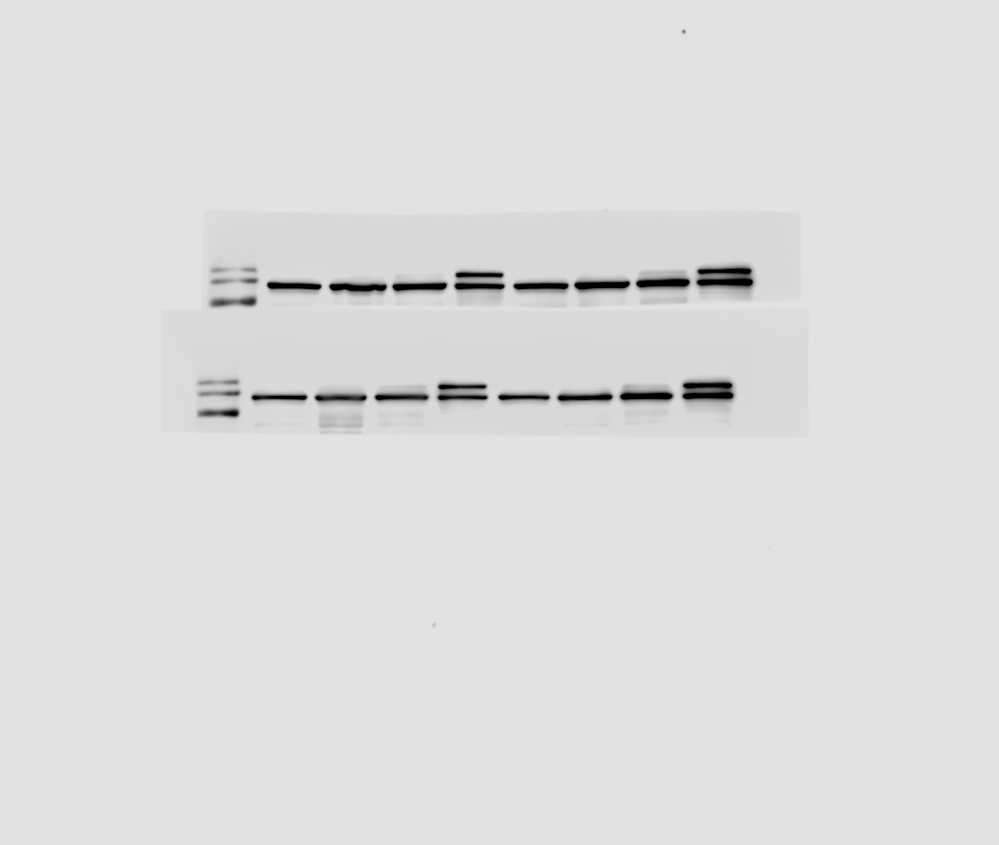

Supplement: Supplementary file 2 [file DataSheet_1.zip › WB original scans/Figure S2 scan 01 (lower membrane).jpg]

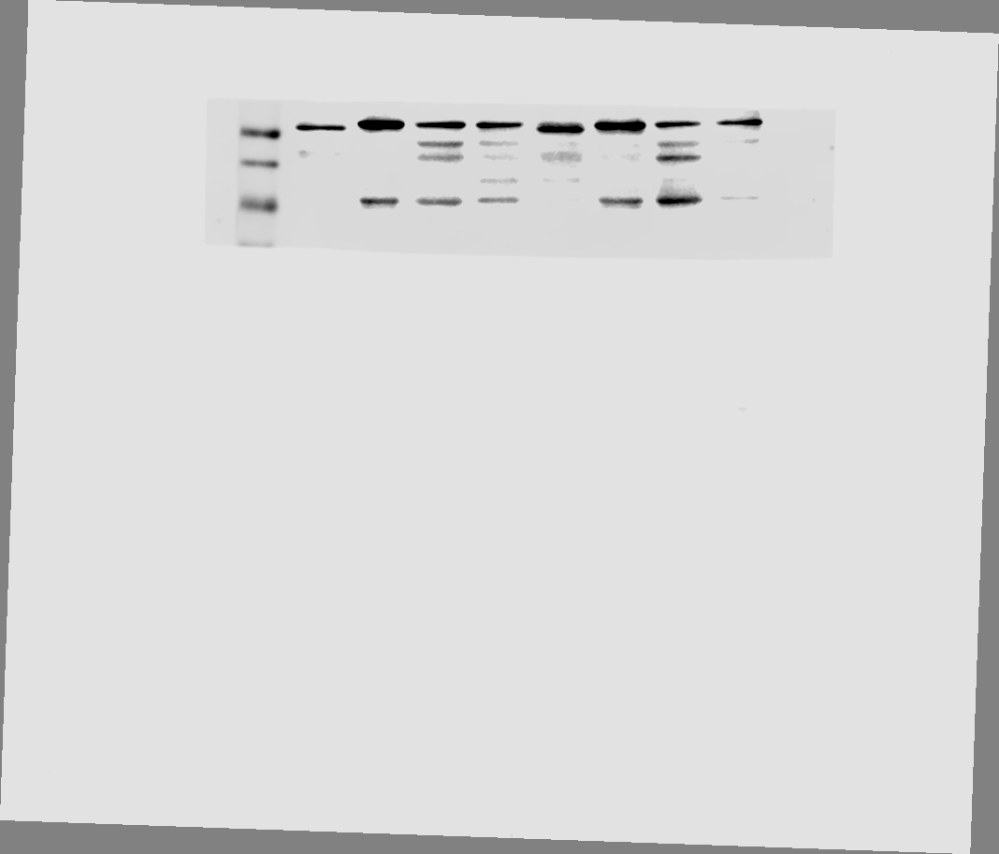

Supplement: Supplementary file 2 [file DataSheet_1.zip › WB original scans/Figure S2 scan 02.jpg]

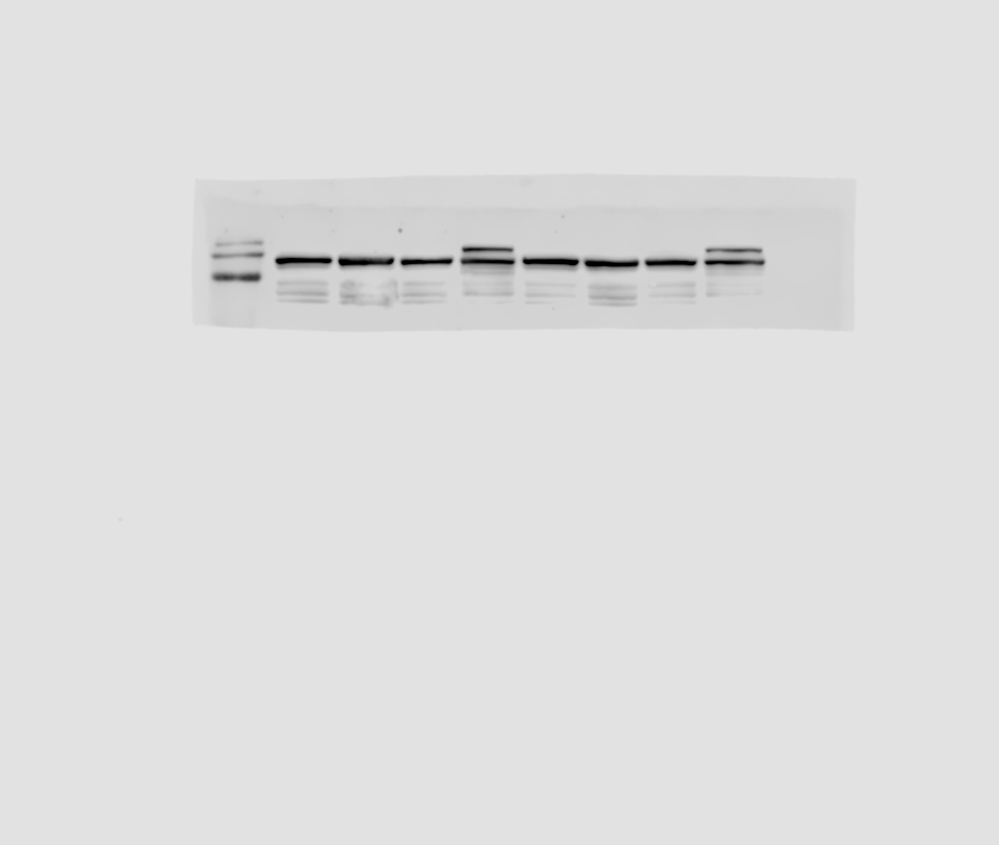

Supplement: Supplementary file 2 [file DataSheet_1.zip › WB original scans/Figure S2 scan 03.jpg]

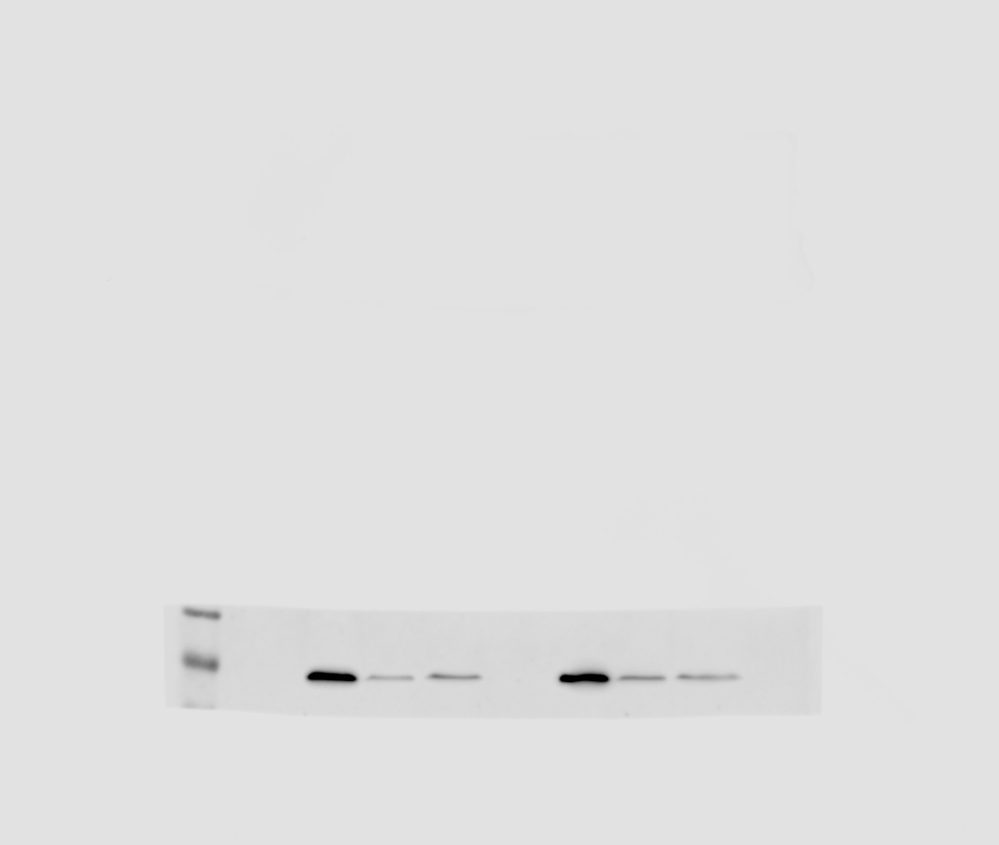

Supplement: Supplementary file 2 [file DataSheet_1.zip › WB original scans/Figure S2 scan 04.jpg]

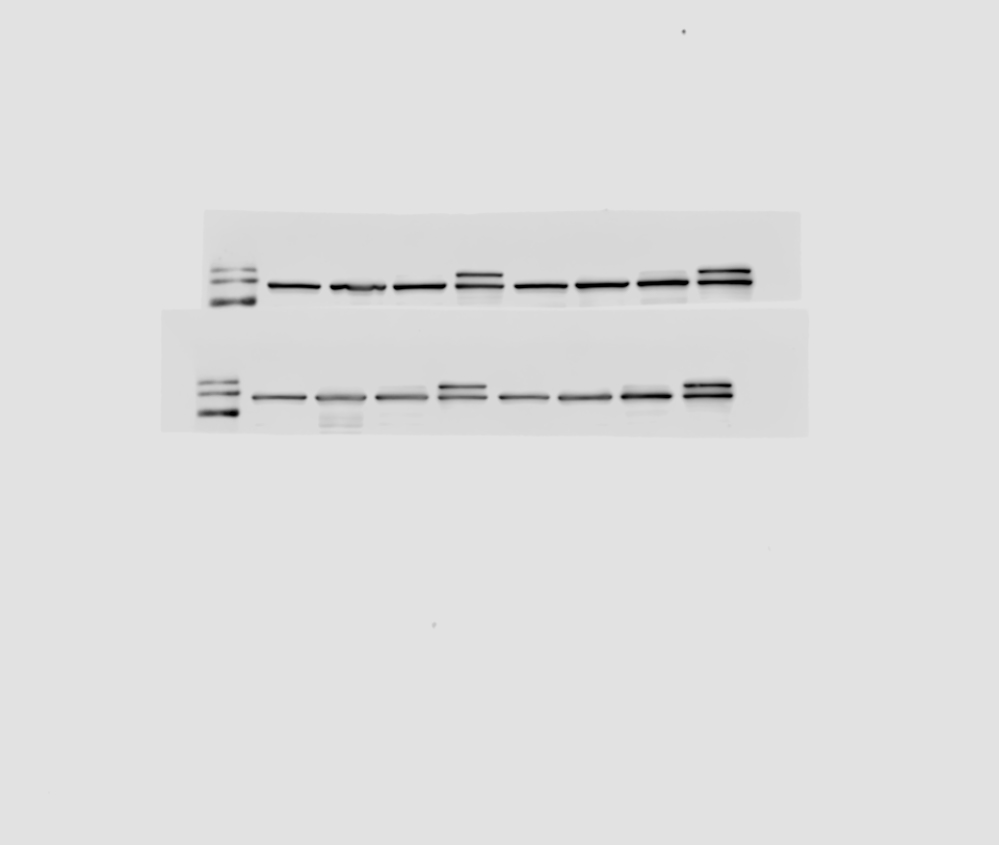

Supplement: Supplementary file 2 [file DataSheet_1.zip › WB original scans/Figure S2 scan 05 (upper membrane).jpg]

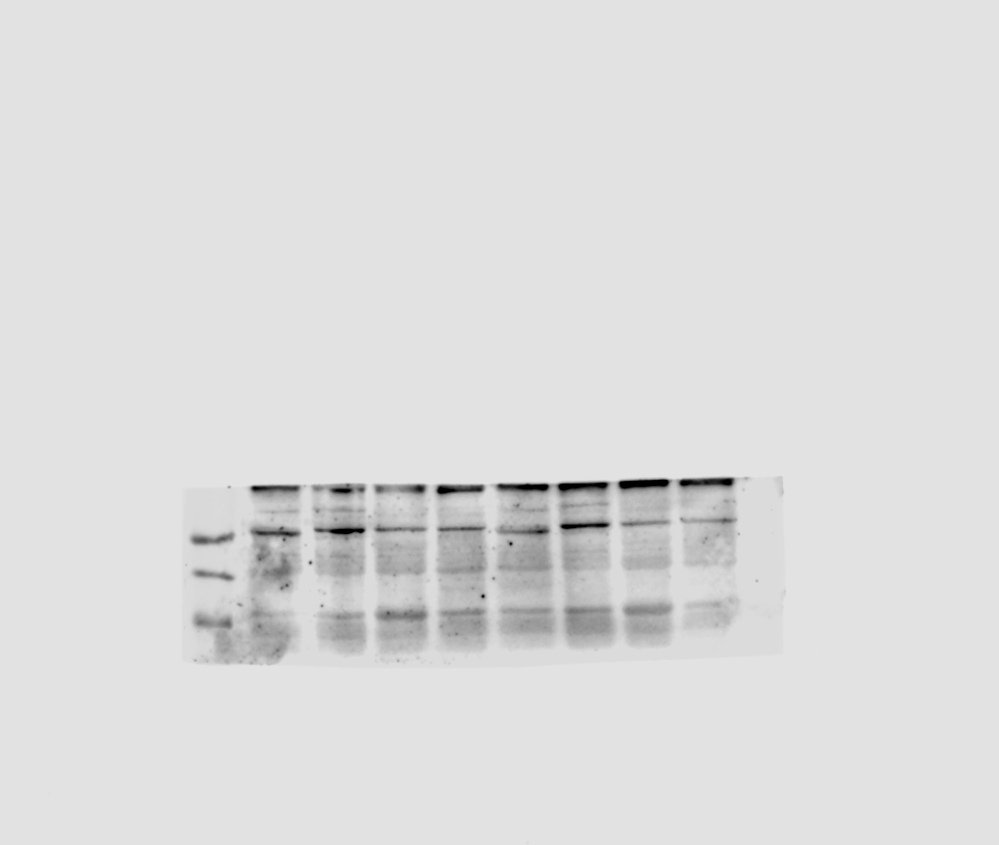

Supplement: Supplementary file 2 [file DataSheet_1.zip › WB original scans/Figure S2 Scan 06.jpg]
